# HBV core protein allosteric modulators differentially alter cccDNA biosynthesis from *de novo* infection and intracellular amplification pathways

**DOI:** 10.1371/journal.ppat.1006658

**Published:** 2017-09-25

**Authors:** Fang Guo, Qiong Zhao, Muhammad Sheraz, Junjun Cheng, Yonghe Qi, Qing Su, Andrea Cuconati, Lai Wei, Yanming Du, Wenhui Li, Jinhong Chang, Ju-Tao Guo

**Affiliations:** 1 Baruch S. Blumberg Institute, Doylestown, Pennsylvania, United States of America; 2 Microbiology and Immunology graduate program, Drexel University College of Medicine, Philadelphia, Pennsylvania, United States of America; 3 National Institute of Biological Sciences, Beijing, China; 4 Arbutus Biopharma Inc., Doylestown, Pennsylvania, United States of America; 5 Hepatology Institute, Peking University People’s Hospital, Beijing, China; University of California, San Diego, UNITED STATES

## Abstract

Hepatitis B virus (HBV) core protein assembles viral pre-genomic (pg) RNA and DNA polymerase into nucleocapsids for reverse transcriptional DNA replication to take place. Several chemotypes of small molecules, including heteroaryldihydropyrimidines (HAPs) and sulfamoylbenzamides (SBAs), have been discovered to allosterically modulate core protein structure and consequentially alter the kinetics and pathway of core protein assembly, resulting in formation of irregularly-shaped core protein aggregates or “empty” capsids devoid of pre-genomic RNA and viral DNA polymerase. Interestingly, in addition to inhibiting nucleocapsid assembly and subsequent viral genome replication, we have now demonstrated that HAPs and SBAs differentially modulate the biosynthesis of covalently closed circular (ccc) DNA from *de novo* infection and intracellular amplification pathways by inducing disassembly of nucleocapsids derived from virions as well as double-stranded DNA-containing progeny nucleocapsids in the cytoplasm. Specifically, the mistimed cuing of nucleocapsid uncoating prevents cccDNA formation during *de novo* infection of hepatocytes, while transiently accelerating cccDNA synthesis from cytoplasmic progeny nucleocapsids. Our studies indicate that elongation of positive-stranded DNA induces structural changes of nucleocapsids, which confers ability of mature nucleocapsids to bind CpAMs and triggers its disassembly. Understanding the molecular mechanism underlying the dual effects of the core protein allosteric modulators on nucleocapsid assembly and disassembly will facilitate the discovery of novel core protein-targeting antiviral agents that can more efficiently suppress cccDNA synthesis and cure chronic hepatitis B.

## Author summary

Persistent HBV infection relies on stable maintenance of a nuclear episomal viral genome called covalently closed circular (ccc) DNA, the sole transcriptional template supporting viral replication. The currently available antiviral therapeutics fail to cure chronic HBV infection due to their failure to eradicate or inactivate cccDNA. In addition to packaging viral pregenomic (pg) RNA and DNA polymerase complex into nucleocapsids for reverse transcriptional DNA replication to take place, HBV core protein also participates in and regulates virion particle assembly, capsid uncoating and cccDNA formation. We report herein an intriguing observation that selected core protein allosteric modulators not only inhibit nucleocapsid assembly, but can also act on assembled, nucleus-bound nucleocapsids to promote their uncoating and consequentially interfere with cccDNA biosynthesis. This finding establishes molecular basis for development of novel core protein targeting antiviral agents with improved efficacy of suppressing cccDNA synthesis and curing chronic HBV infection.

## Introduction

Hepatitis B virus (HBV) is a small DNA virus that chronically infects 240 million people worldwide and causes approximately 686,000 deaths annually due to various severe liver diseases, including cirrhosis, hepatocellular carcinoma (HCC) and liver failure [1]. Currently approved direct-acting antiviral agents against HBV are six nucleos(t)ide analogues that inhibit viral DNA polymerase with varying potency and barriers to drug resistance [2]. Although those viral DNA polymerase inhibitors significantly reduce viral load and prevent liver disease progression, they rarely cure HBV infection due to their inability to eradicate cccDNA [3].

HBV core protein is a small polypeptide of 183 amino acid residues. It exists in infected hepatocytes as several distinct quaternary structures and plays multiple roles in the viral replication cycle [4]. The best characterized function of core protein is the assembly of pre-genomic (pg) RNA and viral DNA polymerase complex into nucleocapsids where HBV DNA synthesis takes place [5]. Moreover, temporally and spatially regulated disassembly (or uncoating) of nucleocapsids is essential for delivery of viral relaxed circular (rc) genomic DNA into the nuclei of infected hepatocytes [6,7], where it is converted to covalently closed circular (ccc) DNA [8,9]. Furthermore, it has also been suggested that core proteins may associate with cccDNA minichromosomes, in an as-yet undefined structural manner, to regulate its transcription [10]. Interestingly, it was also reported that core protein can be hijacked by host immune responses to recruit cytokine-induced DNA cytosine deaminase APOBEC3A to cccDNA minichromosomes, which results in cytosine deamination and decay of cccDNA [11,12].

Due to their unique structures and essential roles in viral replication, disruption of, or interference with, nucleocapsid assembly and/or disassembly with small molecular core protein allosteric modulators (CpAMs) represents a new frontier in development of novel antiviral agents against HBV [13,14]. Over the last two decades, at least five chemotypes of CpAMs have been reported [13]. Those compounds bind to a hydrophobic pocket, designated as the HAP pocket, at the dimer-dimer interface near the C-termini of core protein subunits [15,16]. Binding of these molecules in the HAP pocket induces large scale allosteric conformational changes in core protein subunits and alters the capsid assembly kinetics and pathways [4,17]. While heteroaryldihydropyrimidines (HAPs), such as Bay 41–4109 and GLS4, misdirect capsid assembly to form non-capsid polymers of core proteins [17,18], all other chemotypes of CpAMs, including sulfamoylbenzamides (SBAs) and phenylpropenamides (PPAs), represented by ENAN-34017 and AT-61, respectively (S1 Fig), induce the formation of morphologically “normal” empty capsids with distinct quaternary and/or tertiary structural changes and thus, preclude viral DNA replication [19,20]. Thus far, several HAPs and SBAs have been shown to inhibit HBV replication in animal models and are currently under preclinical or clinical development [14,21].

Inspired by the observation that a small molecule compound targeting the capsid protein of dengue virus has dual effects on both the assembly and disassembly (or uncoating) of the viral capsids [22], we hypothesized that HBV CpAMs may not only disrupt capsid assembly, but also alter the structure and function of assembled nucleocapsids and consequentially affect viral DNA replication and/or cccDNA synthesis. Indeed, we have now obtained evidence showing that HAPs and SBAs, but not PPAs, induce disassembly of nucleocapsids from virions as well as double-stranded DNA-containing cytoplasmic progeny nucleocapsids and consequentially interfere with cccDNA biosynthesis from *de novo* infection and intracellular amplification pathways.

## Results

### CpAMs inhibit *de novo* cccDNA synthesis

**Fig 1:**
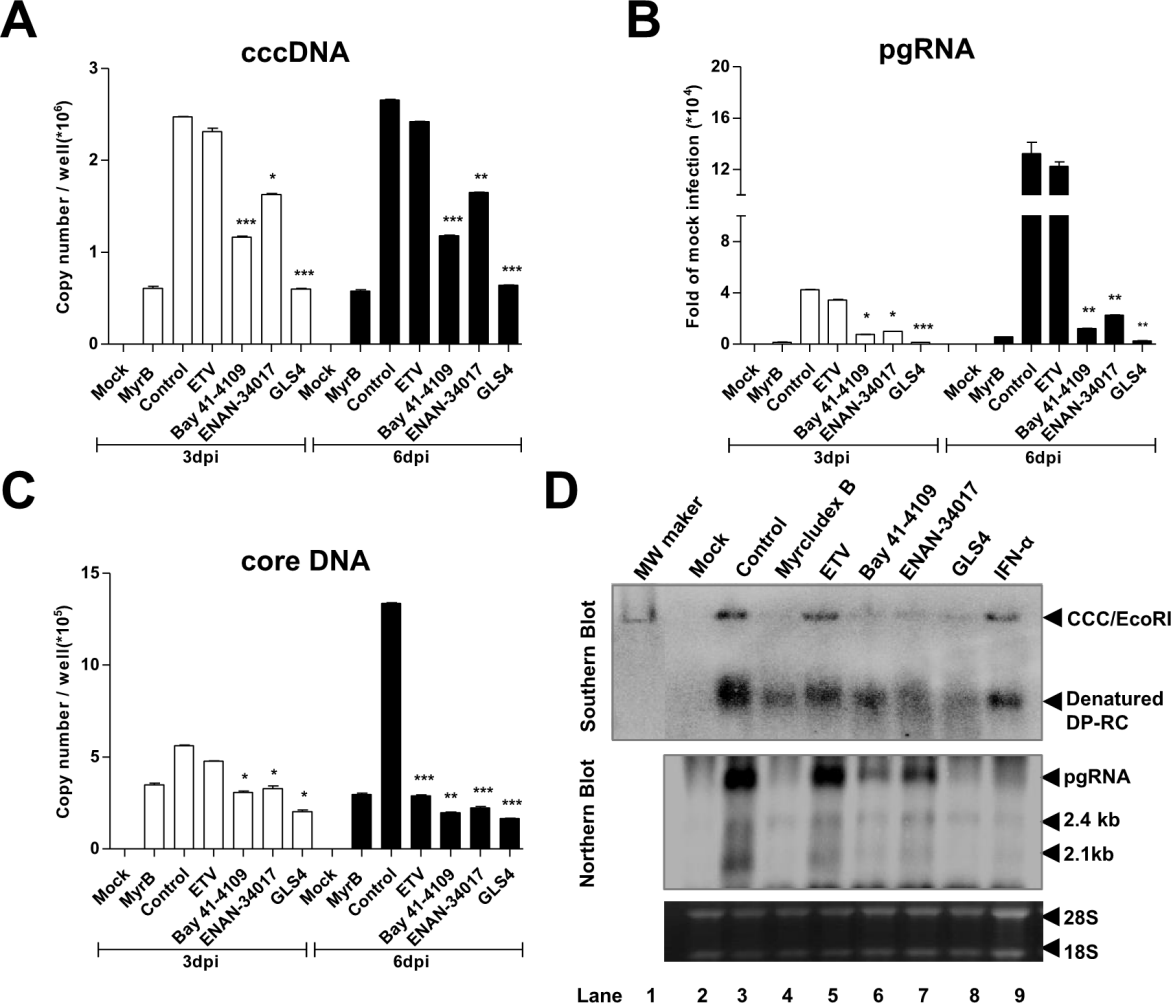
HBV capsid assembly modulators efficiently inhibit cccDNA formation during *de novo* HBV infection of C3A^hNTCP^ cells. C3A^hNTCP^ cells were infected with HBV at an MOI of 500 genome equivalents. The cells were mock-treated or treated with 100nM of MyrB, 1 μM of ETV, 2.5 μM of Bay 41–4109, 1 μM of GLS-4, 5 μM of ENAN-34017 or 1,000 IU/ml of IFN-α, starting from 24 h before infection until harvesting at 3 or 6 days post infection (dpi). HBV cccDNA **(A)**, pgRNA **(B)** and cytoplasmic core DNA **(C)** were quantified by real-time PCR assays. Differences in viral cccDNA, core DNA or pgRNA between mock-treated control and treated groups were statistically analyzed (t-test, * *p* <0.05, ** *p* <0.01, *** *p* <0.001). **(D)** Hybridization analyses of HBV replication intermediates in cells harvested at 6 dpi. *Upper pan*e*l*, Hirt DNA extracted from the cells harvested at 6 dpi were denatured at 88°C for 5 min to denature DP-rcDNA to single-stranded DNA and followed by restriction with EcoRI to convert cccDNA into unit-length double stranded linear DNA and detected by Southern blot hybridization (labeled as CCC/EcoRI). Unit-length HBV linear DNA served as a molecular weight marker. *Lower panel*, HBV RNAs, pre-genomic RNA (pgRNA), 2.4 and 2.1kb mRNA specifying envelope proteins were determined by Northern blot hybridization. 28S and 18S ribosomal RNA (rRNA) served as loading controls.

Discovery of human sodium taurocholate cotransporting polypeptide (hNTCP) as the *bona fide* receptor for HBV infection of hepatocytes allows for establishment of convenient HepG2-derived HBV infection cell culture systems [23,24]. We have thus established a novel NTCP-expressing human cell line, designated as C3A^hNTCP^, and demonstrated its susceptibility to HBV infection. The parental cell line, C3A, is a subclone of HepG2 cells that exhibits strong contact inhibition of growth and metabolic features that are more similar to normal hepatocytes [25]. As shown in S2 Fig, cccDNA became detectable as early as 1 day and reached maximum levels at 2 day post infection by qPCR and conventional Southern blot assays. HBV pgRNA and core-associated viral DNA replication intermediates as well as core protein also accumulated sequentially in infected cultures. HBsAg was readily detectable by ELISA as a product of HBV infection in the culture media. In order to investigate the effects of CpAMs on HBV infection, particularly cccDNA synthesis from a *de novo* infection, C3A^hNTCP^ cells were mock-treated or treated with representative HAP (Bay 41–4109, GLS4) or SBA (ENAN-34017) and control compounds entecavir (ETV) or Myrcludex B (MyrB), starting at 24 h before HBV infection until harvesting at day 3 and day 6 post infection. As expected (Fig 1), MyrB, an acylated peptide derived from the HBV large envelope protein blocks virus entry [26], inhibited cccDNA formation and consequential accumulation of pgRNA and core DNA. Also as anticipated, ETV, an HBV DNA polymerase inhibitor, did not affect the synthesis of cccDNA and accumulation of pgRNA, but inhibited the synthesis of core DNA [27]. Interestingly, GLS4, Bay 41–4109 and ENAN-34107 significantly reduced the amounts of cccDNA. Viral pgRNA and core DNA were also proportionally reduced. A more detailed time course study spanning the first four days post infection revealed that the three CpAMs significantly inhibited cccDNA formation, whereas ETV and IFN-α did not (S3 Fig) (Fig 1D, lanes 5 and 9). However, while ETV did not reduce viral RNA, IFN-α reduced the levels of viral pgRNA and 2.4/2.1 kb mRNA, presumably due to suppression of cccDNA transcription [28,29].

**Fig 2:**
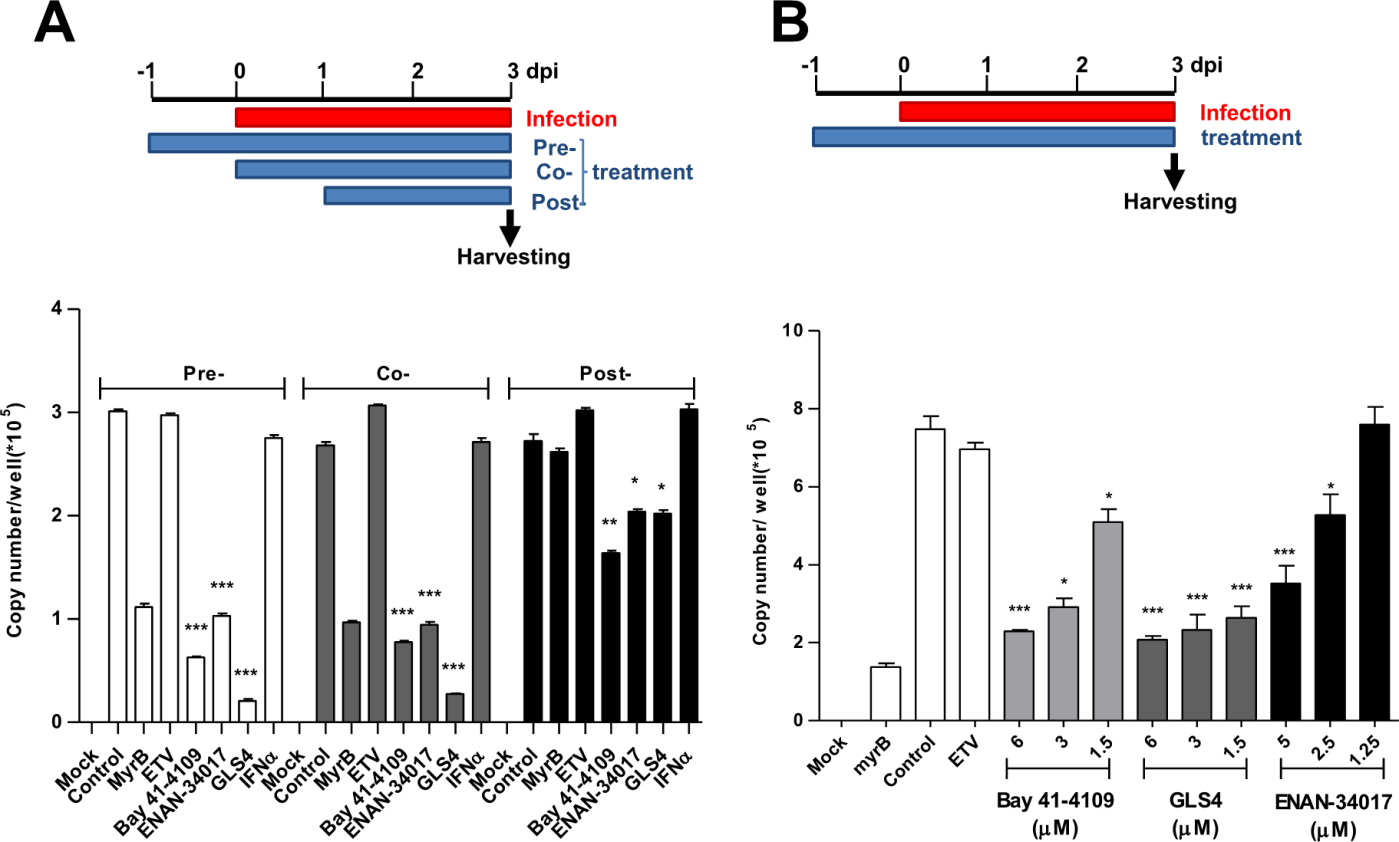
Inhibition of cccDNA formation by HBV capsid assembly modulators is time- and concentration-dependent. **(A)** C3A^hNTCP^ cells were infected with HBV at an MOI of 500 genome equivalents. The cells were mock-treated or treated with 100 nM of MyrB, 1 μM of ETV, 2.5 μM of Bay 41–4109, 1 μM of GLS-4, 5 μM of ENAN-34017 or 1,000 IU/ml of IFN-α., starting from 24 h before infection, at infection or 24 h post infection until harvesting at 3 days post infection. HBV cccDNA was quantified by a real-time PCR assay. **(B)** C3A^hNTCP^ cells were infected with HBV at an MOI of 500 genome equivalents. The cells were mock-treated or treated with the indicated concentrations of Bay 41–4109, GLS4 or ENAN-34017, starting from 24 h before infection until harvesting at 3 days post infection. HBV cccDNA were quantified by a real-time PCR assay. Differences in viral cccDNA between mock-treated control and treated group cultures under each treatment schedule were statistically analyzed (t-test, * *p* <0.05, ** *p* <0.01, *** *p* <0.001).

To further characterize the inhibitory effect of CpAMs on cccDNA biosynthesis, time-of-addition and dose response experiments were performed. In agreement with its mode of action, MyrB treatment starting at 24 h before or at the time of infection efficiently blocked cccDNA formation, whereas delayed treatment starting at 24 h post infection completely failed to inhibit cccDNA formation (Fig 2A). Consistent with the kinetics of cccDNA formation in this cell culture system (S3 Fig), while CpAM treatment starting at 24 h before or at the time of infection reduced cccDNA formation at similar efficiency, their inhibitory effects were significantly reduced when the treatment started at 24 h post infection. Moreover, all the three CpAMs inhibited cccDNA formation in a concentration dependent manner (Fig 2B). Those results are in agreement with a report published during the preparation of this manuscript that Bay 41–4109 and SBA derivative JNJ-632 inhibited HBV cccDNA synthesis during HBV infection of human primary hepatocytes [30].

To investigate the possibility that the observed inhibition of CpAMs on cccDNA formation is due to inhibition of NTCP-mediated HBV entry, we examined their effects on hepatitis D virus (HDV) infection of C3A^hNTCP^ cells. The results demonstrated that while MyrB efficiently reduced genomic and antigenomic HDV RNA by more than 3 log, ETV and IFN-α as well as Bay-41-4109 and ENAN-34017 did not apparently alter the amounts of HDV RNA in the infected cultures (S4 Fig). The results thus imply that inhibition of cccDNA synthesis by the CpAMs is due to their interaction with HBV-specific components, most possibly nucleocapsids, but not the host cellular factor(s) in the entry pathway shared by HBV and HDV.

### CpAMs accelerate intracellular cccDNA synthesis from cytoplasmic progeny nucleocapsid rcDNA

**Fig 3:**
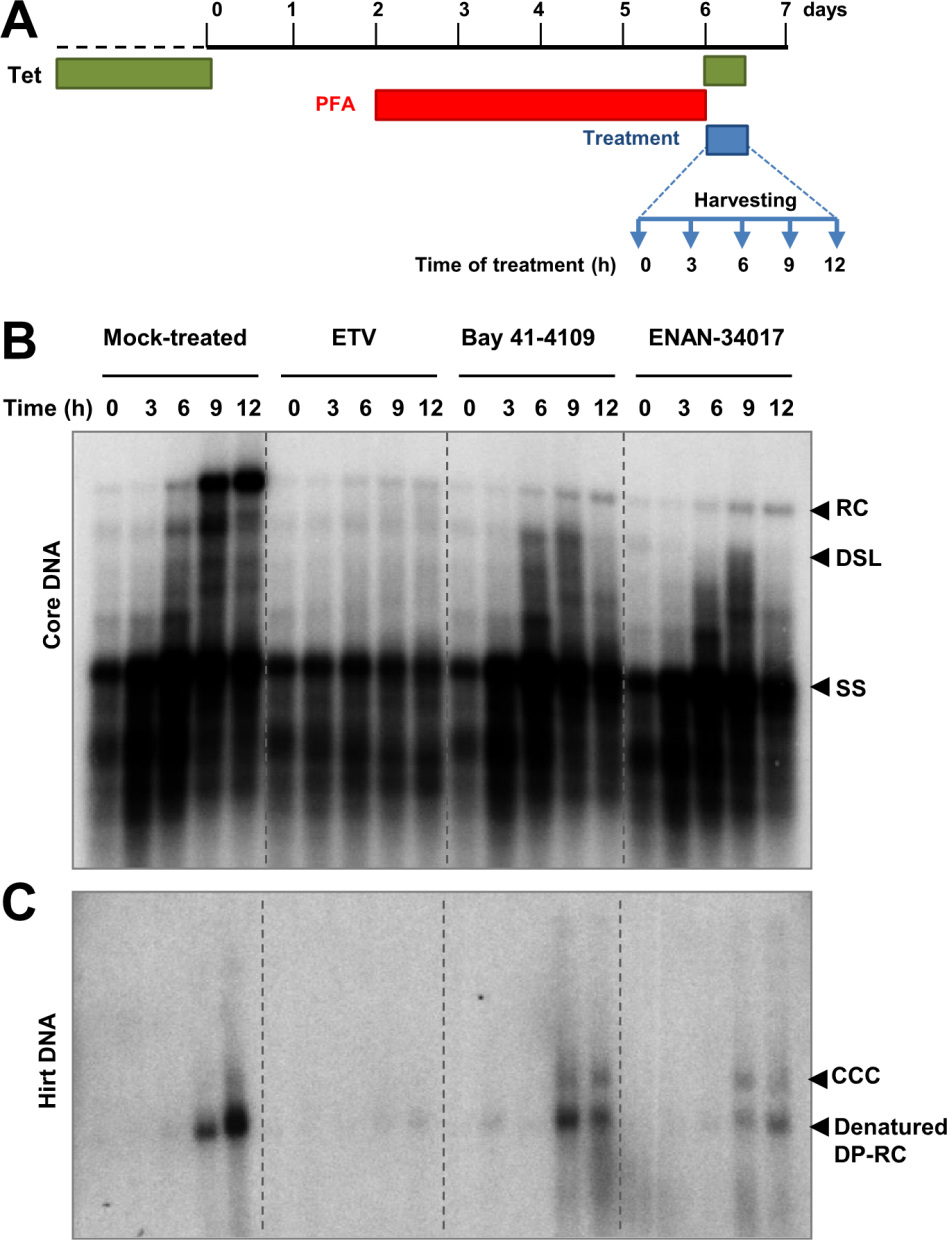
HBV capsid assembly modulators accelerate cccDNA formation through the intracellular amplification pathway. As depicted in panel **(A),** HepAD38 cells were treated with 2 mM of PFA from day 2 to day 6 after removal of tet from medium. On day 6, PFA-containing medium were removed and the cells were mock-treated or treated with 1 μM of ETV, 2.5 μM of Bay 41–4109 or 5 μM of ENAN-34017 and harvested at 0, 3, 6, 9 and 12 h of the treatment. The cytoplasmic core DNA **(B)**, cccDNA and DP-rcDNA **(C)** were analyzed by Southern blot hybridization. The relaxed circular (RC) DNA, double-stranded linear (DSL) DNA and full-length single stranded (SS) DNA species were indicated. For cccDNA and DP-rcDNA analysis, before loading, Hirt DNA were denatured at 88°C for 5 min, which denatures DP-rcDNA into single-stranded DNA (labeled as denatured DP-rc).

**Fig 4:**
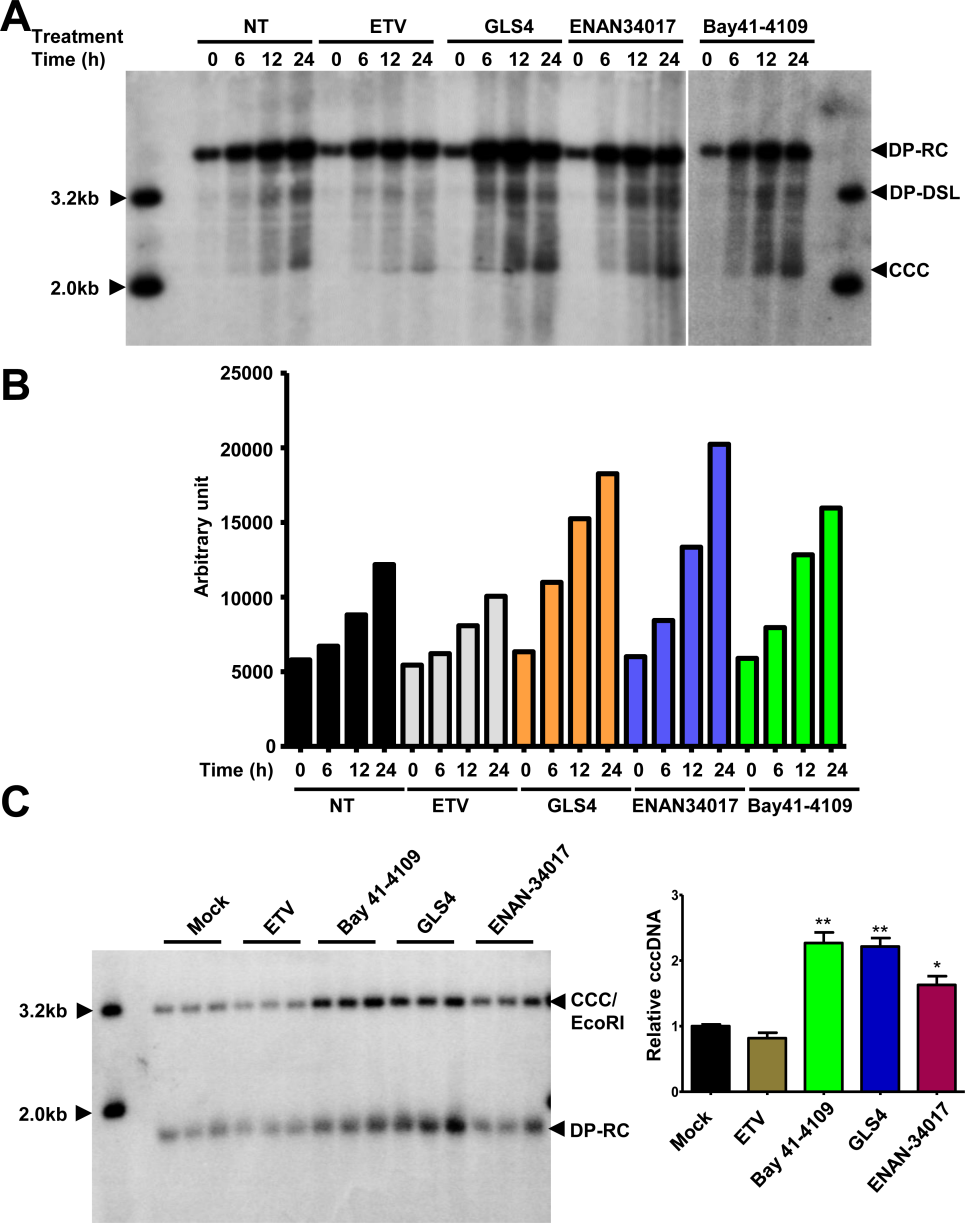
Quantitative analyses of the effects of CpAMs on cccDNA synthesis. HepAD38 cells were treated with 2 mM of PFA from day 2 to day 6 after removal of tet from medium. On day 6, PFA-containing medium were removed and the cells were mock-treated or treated with 1 μM of ETV, 1 μM of GLS4, 2.5 μM of Bay 41–4109 or 5 μM of ENAN-34017 and harvested at 0, 6, 12 and 24 h of treatment. (**A**) Hirt DNA were extracted and resolved in 1.5% agarose gel electrophoresis. HBV DNA were detected by Southern blot hybridization. (**B**) The amounts of cccDNA in each sample were quantified with Tyhoon FLA 7000 and plotted as arbitrary units. **(C)** Hirt DNA extracted from cells treated with the indicated compounds for 12 h after PFA removal were denatured at 88°C for 5 min and followed by EcoRI digestion. The DNA samples were then resolved in agarose gel and HBV DNA were detected by Southern blot hybridization (left panel). The amounts of cccDNA in each samples were quantified and normalized to the levels of mock-treated controls. The relative amounts of cccDNA were plotted (right panel). The relaxed circular (RC) DNA, double-stranded linear (DSL) DNA and cccDNA (CCC) species were indicated. * *p* < 0.05, ** *p* < 0.01 (by t-test).

Besides being synthesized from incoming virion DNA in the *de novo* infection, cccDNA in infected hepatocytes can also be amplified by delivery of rcDNA from cytoplasmic progeny nucleocapsids into the nucleus and conversion to cccDNA [31–33]. This intracellular cccDNA amplification pathway has been demonstrated to function in cultured cells and *in vivo* in duck hepatitis B virus (DHBV)-infected ducks, and is regulated by the large envelope protein and host cellular factors [33,34]. Because CpAMs inhibit pgRNA encapsidation and thus preclude viral DNA replication and intracellular amplification of cccDNA, their effects on intracellular progeny nucleocapsids and cccDNA amplification cannot be investigated in cells directly treated with those compounds [35]. To circumvent this problem, as depicted in Fig 3A, we first arrested HBV DNA replication by culturing HepAD38 cells in medium without tetracycline, but containing the reversible viral DNA polymerase inhibitor Foscarnet (trisodium phosphonoformate, or PFA for short) for four days, which arrested HBV DNA replication predominantly at the stages of incomplete and complete minus-strand DNA (Fig 3B) [36]. The cells were then cultured in the presence of tetracycline to stop HBV pgRNA transcription from integrated transgene in cellular chromosome, and also in the absence of PFA to allow viral DNA replication and cccDNA synthesis to resume. At the time of PFA withdrawal, mock treatment or treatment with ETV, Bay-41-4109, ENAN-34017 was initiated and cells were harvested at the indicated time points for analyses of HBV core DNA and cccDNA. Synthesis and identity of cccDNA in this cell culture system were confirmed by its resistance to heat denaturing and conversion into 3.2kb unit-length DNA after heat denaturing and EcoRI digestion (S5 Fig). As shown in Fig 3B, upon removal of PFA from culture medium, the incomplete and complete minus strand, partial double-strand and complete double strand HBV DNA species (including rcDNA) sequentially increased from 3 to 12 h (Fig 3B). Deproteinized rcDNA (DP-rcDNA) and cccDNA became readily detectable at 9 and 12 h, respectively (Fig 3C). As expected, ETV treatment inhibited the elongation of arrested HBV DNA species and prevented the production of rcDNA as well as DP-rcDNA and cccDNA. Interestingly, although Bay-41-4109 or ENAN-34017 treatment did not inhibit elongation of minus-strand and partial double-strand DNA in the cytoplasmic nucleocapsids, the rcDNA was not accumulated in those cells (Fig 3B). However, analysis of Hirt DNA indicated that the amount of DP-rcDNA was not apparently reduced by CpAM treatment and to our surprise, the cccDNA was readily detected as early as 9 h after the removal of PFA in CpAM-treated cells. Experiments with additional CpAMs and extended treatment duration further supported the notion that the selected HAP and SBA compounds reduced the accumulation of cytoplasmic rcDNA-containing nucleocapsids (S6 Fig), but significantly accelerated cccDNA synthesis from intracellular progeny nucleocapsids, as demonstrated by two different cccDNA assay procedures (S6 Fig and Fig 4).

### CpAMs do not inhibit completion of positive strand DNA synthesis, but confer DNase I sensitivity of mature forms of viral DNA

**Fig 5:**
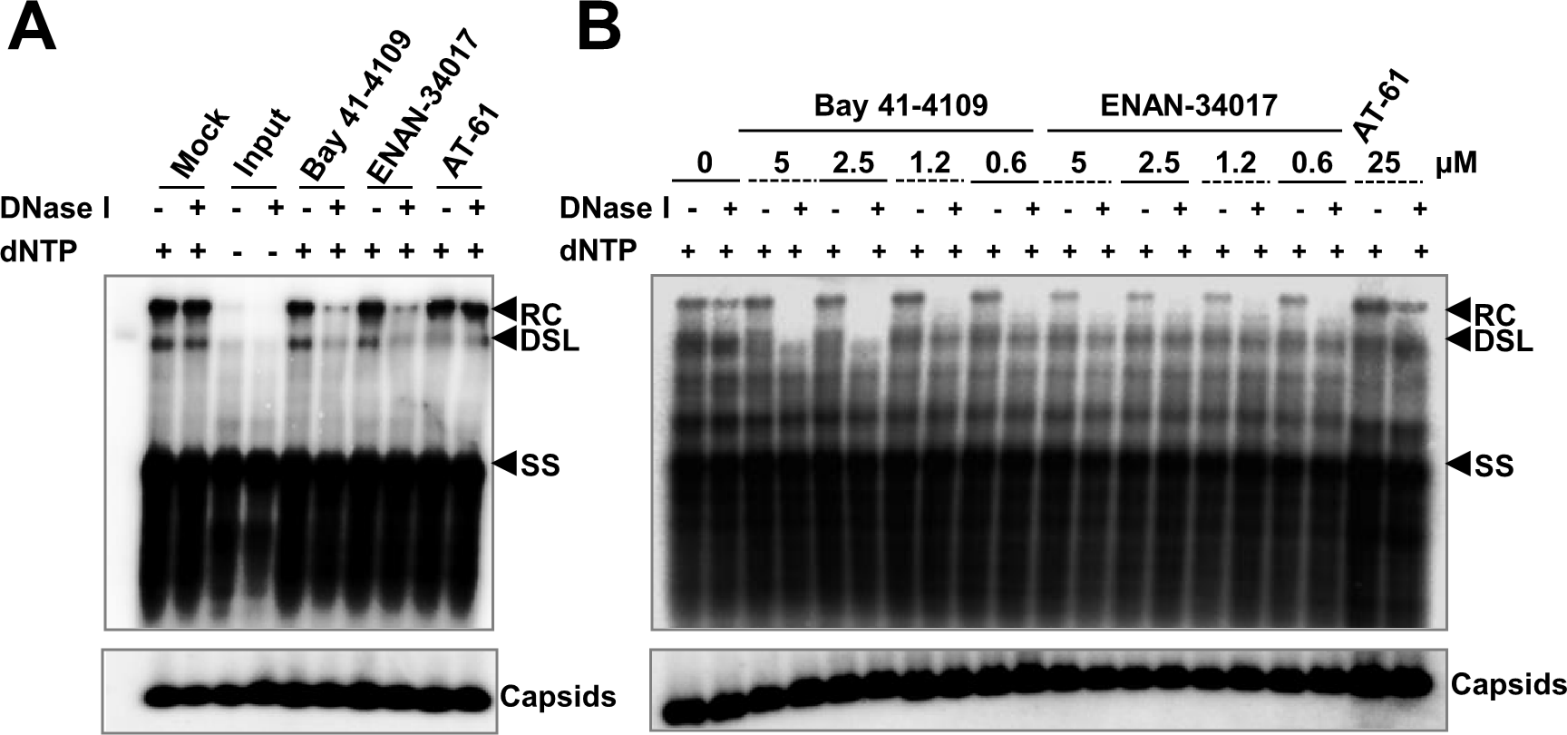
HBV capsid assembly inhibitors do not inhibit completion of positive-strand DNA synthesis, but render the mature forms of HBV DNA sensitive to DNase I digestion. HepAD38 cells were treated with 2 mM of PFA from day 2 to day 6 post removal of tet from medium. Intracellular capsids were purified by sucrose gradient centrifugation. **(A)** Endogenous DNA polymerase reactions with the purified HBV capsids were performed in the absence or presence of dNTP, 2.5 μM of Bay 41–4109, 5 μM of ENAN-34017 or 25 μM of AT-61 at 37°C for 16 h. Three-fourths of each reaction were subjected for extraction of viral DNA without or with prior DNase I digestion at 37°C for 30 min. The viral DNA were detected by Southern blot hybridization with full-length riboprobe recognizing minus strand DNA. The remaining one-fourth of each reaction was analyzed by a particle gel assay to detect HBV capsids. **(B)** Endogenous DNA polymerase reactions were performed in the presence of dNTP and indicated concentrations of Bay 41–4109, ENAN-34017 or AT-61 at 37°C for 16 h. Viral DNA and capsids were analyzed as described above. The relaxed circular (RC) DNA, double-stranded linear (DSL) DNA and full-length single stranded (SS) DNA species were indicated.

The apparent contradicting effects of CpAMs on cytoplasmic rcDNA-containing nucleocapsid accumulation and kinetics of cccDNA synthesis in HepAD38 cells prompted us to investigate whether those compounds inhibited the completion of rcDNA synthesis and/or destabilized the nucleocapsids containing mature forms of double-stranded DNA. This latter possibility may explain the observed acceleration of cccDNA formation from intracellular progeny nucleocapsids [7,37]. To this end, HBV capsids were purified from the cytoplasmic fraction of PFA-treated cells and an endogenous DNA polymerase assay were performed *in vitro* in the absence or presence of CpAMs. To probe the integrity of nucleocapsids, the accessibility of viral DNA to DNase I digestion were tested at the completion of endogenous DNA polymerase reaction, but before extraction of viral DNA. Southern blot analysis of viral DNA species indicated that rcDNA can be efficiently synthesized in the *in vitro* endogenous DNA polymerase reaction when dNTPs were provided (Fig 5A). Furthermore, the presence of Bay 41–4109, ENAN-34017 or AT-61 did not inhibit rcDNA synthesis. However, the rcDNA synthesized in the presence of Bay 41–4109 and ENAN-34017, but not AT-61, was susceptible to DNase I digestion (Fig 5A and 5B). Hence, our results indicate that CpAMs do not inhibit HBV DNA synthesis, but favor a hypothesis that the selected CpAMs specifically alter the structure of, and destabilize mature rcDNA-containing nucleocapsids and facilitate rcDNA nuclear delivery and synthesis of cccDNA. Because the mature rcDNA-containing nucleocapsids only constitute a small fraction of total cytoplasmic capsids [38], it is not surprise that the CpAM treatment did not alter the amounts and migration mobility of capsids, as revealed by a native agarose gel electrophoresis-based particle gel assay (Fig 5A and 5B, lower panels) [35,39].

### CpAMs confer susceptibility of virion DNA to DNase I digestion

**Fig 6:**
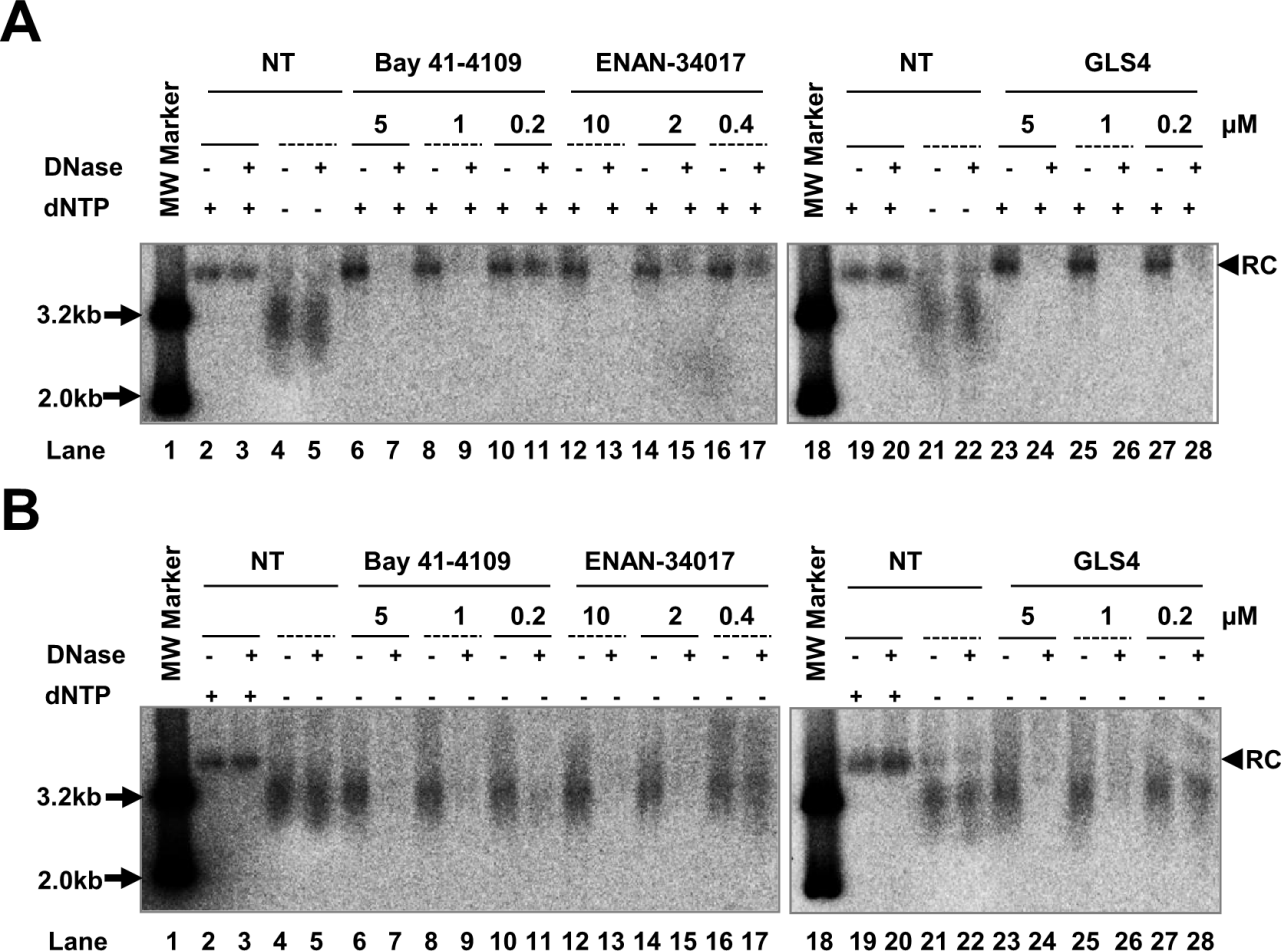
HBV capsid assembly modulator treatment confers HBV virion DNA sensitivity to DNase digestion. **(A)** Each of the endogenous DNA polymerase reaction contains approximately 10^8^ HBV virions prepared from chronic HBV carriers and incubated in the presence or absence of dNTP and indicated concentrations of Bay 41–4109, ENAN-34017 or GLS4 at 37°C for 16 h. Viral DNA were extracted without or with prior DNase I digestion at 37°C for 30 min and detected by Southern blot hybridization with full-length riboprobe recognizing minus strand DNA. **(B)** Approximately 10^8^ HBV virions in each endogenous DNA polymerase reaction were incubated in the absence of dNTP (except for lanes 2, 3, 19 and 20) and absence or presence of the indicated concentrations of Bay 41–4109, ENAN-34017 or GLS4 at 37°C for 16 h. Viral DNA were extracted and analyzed as described above.

The differential effects of CpAMs on cccDNA synthesis from the *de novo* infection and intracellular amplification pathways argues that the compounds may have a different effect on the nucleocapsids in virions and in the cytoplasm. To investigate this possibility, we purified HBV virion particles from the blood of a chronic HBV carrier and examined the effects of CpAMs on rcDNA synthesis and integrity of nucleocapsids in an *in vitro* endogenous DNA polymerase reaction as described above. Similar to the results obtained with cytoplasmic nucleocapsids, the presence of Bay 41–4109, ENAN-34017 or GLS4 did not inhibit rcDNA synthesis from partially double-stranded virion DNA, but conferred susceptibility of virion rcDNA to DNase I digestion in a concentration–dependent manner (Fig 6A). To further investigate whether CpAMs can directly induce structural change of the partially double-stranded DNA-containing nucelocapsids, virion particles prepared from the patient serum were treated with the CpAMs in an endogenous DNA polymerase reaction without dNTP and followed by DNase I treatment prior to DNA extraction. The results showed that each of the three tested CpAMs rendered all virion DNA species susceptible to DNase I digestion in a concentration-dependent manner (Fig 6B). Hence, the results indicate that irrespective to the maturation stage of viral DNA, the nucleocapsids derived from virion particles are sensitive to CpAM-induced structural changes that expose viral DNA for DNase I digestion.

### Elongation of positive-stranded DNA gradually increases the vulnerability of cytoplasmic nucleocapsids to CpAMs

**Fig 7:**
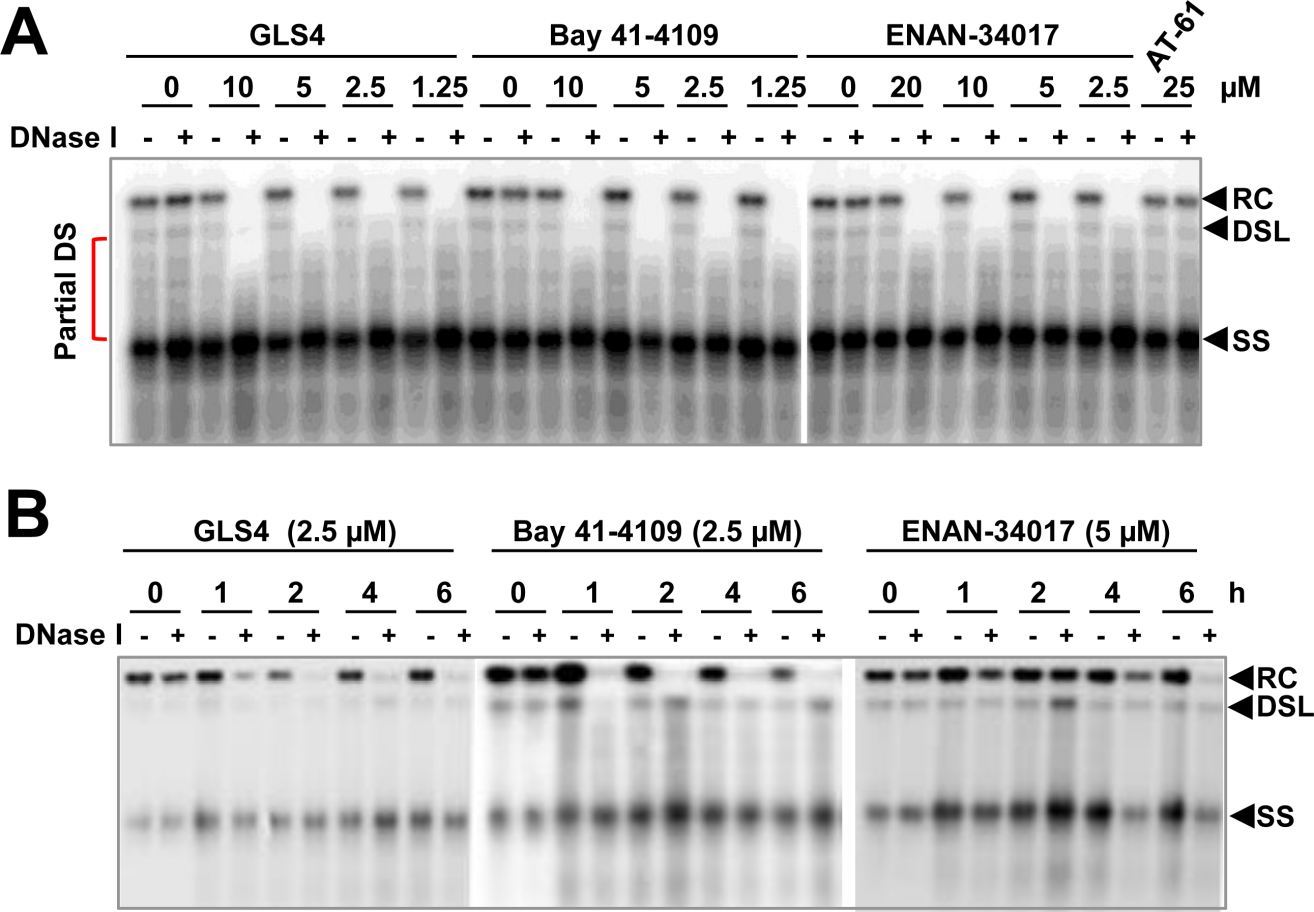
CpAM treatment confers double-stranded DNA in cytoplasmic progeny nucleocapsids sensitive to DNase I digestion in a concentration- and time-dependent manner. HBV capsids purified from HepAD38 cells were mock-treated or treated with the indicated concentrations of GLS4, Bay 41–4109 or ENAN-34017 in reactions containing 150 mM NaCl, 50 mM Tris-HCl, pH8.0, 10 mM MgCl_2_, 1 mM DTT and 0.1% NP-40 at 37°C for 16 h **(A)** or harvested at the indicated time of incubation **(B)**. Viral DNA were extracted without or with prior DNase I digestion at 37°C for 30 min and detected by Southern blot hybridization with a full-length riboprobe that hybridizes to negative strand of HBV DNA. Partial DS: partially double-starnded DNA. Partial DS: partially double stranded DNA.

Encouraged by the observation that the CpAM-induced virion DNA susceptibility to DNase I did not depend on active DNA polymerase reaction or ongoing DNA chain elongation (Fig 6B), we further examined the effects of CpAMs on capsids purified from the cytoplasm of HepAD38 cells in an endogenous DNA polymerase reaction buffer without dNTPs. As shown in Fig 7A, while rcDNA became susceptible to DNase I digestion at lower concentrations of GLS4 or ENAN-34017, the less mature, partially double-stranded DNA species became susceptible to DNase I digestion under gradually increased concentrations. This result strongly suggests that elongation of positive strand DNA in hepatocytes may incrementally induce structural changes of nucleocapsids and confer gradually increased sensitivity to the CpAM induction of viral DNA exposure. Moreover, a comprehensive dose-response experiment revealed that the minimum concentrations of ENAN-34107, Bay 41–4109 and GLS4 that induce a complete exposure of rcDNA are approximately 0.3, 0.04 and <0.02 μM, respectively (S7 Fig), which are comparable to their potency to inhibit nucleocapsid assembly and viral DNA replication in HepG2 cells [35,39,40]. A kinetics study further revealed that while up to 6 h incubation was required for ENAN-34017 to induce significant exposure of rcDNA, 1 to 2 h exposure was sufficient for GLS4 and Bay 41–4109 to induce an extensive exposure of rcDNA (Fig 7B). To determine the kinetics of CpAMs induction of rcDNA exposure in cells, HepAD38 cells cultured in tet-free medium for 6 days were treated with GLS4 for the indicated periods of time. The cytoplasmic lysates were mock-treated or treated with DNase I before extraction of DNA. As shown in S8 Fig, treatment of GLS4 for 6 h induced extensive rcDNA exposure for DNase I digestion.

### CpAMs promote uncoating of mature nucleocapsids

**Fig 8:**
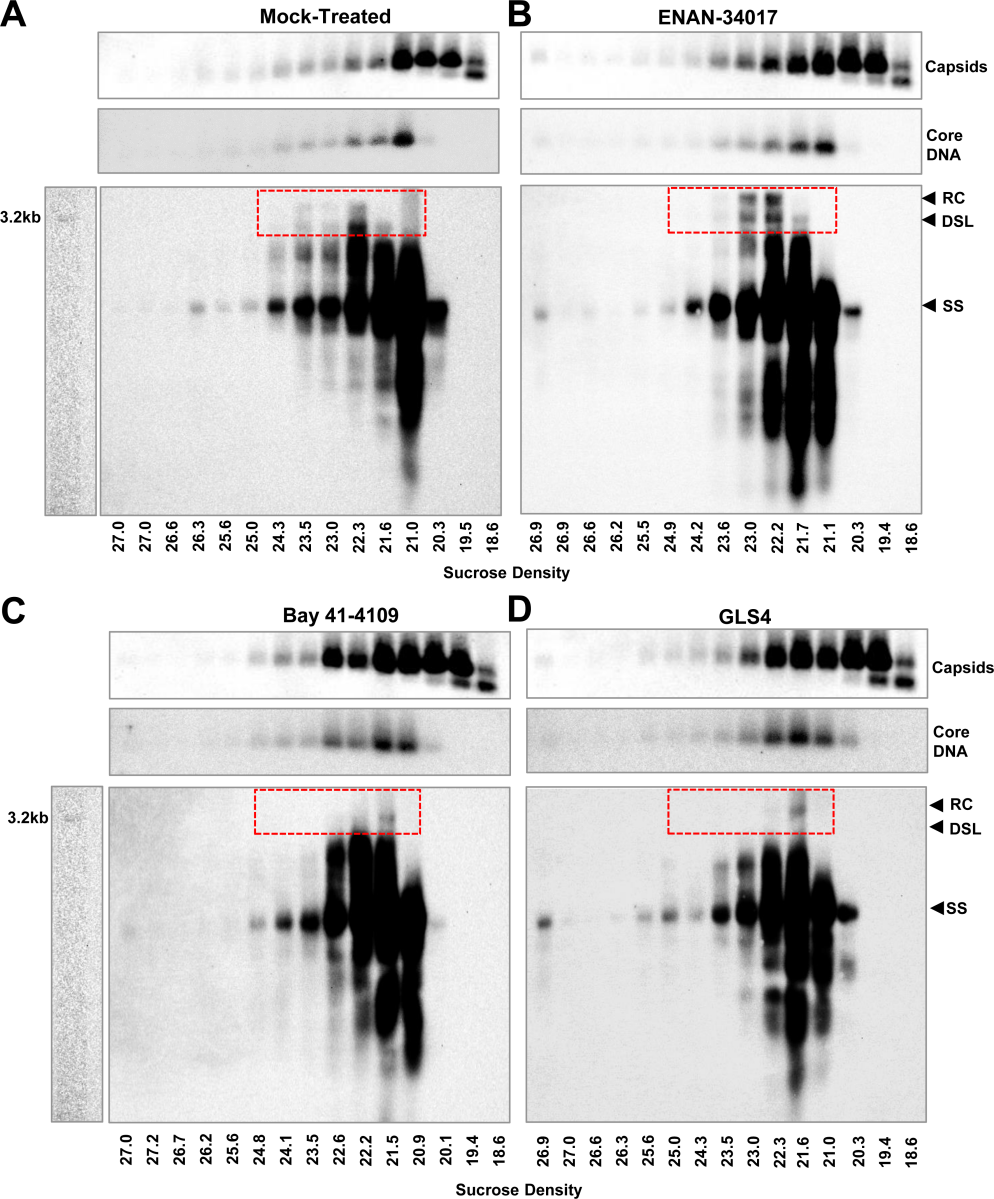
Differential effects of different chemotypes of CpAMs on integrity of mature double-stranded DNA-containing nucleocapsids. HBV capsids prepared from HepAD38 cells were mock treated **(A)** or incubated with 5 μM ENAN-34017 **(B)**, 3 μM Bay 41–4109 **(C)** or 3 μM GLS4 **(D)** in a reaction containing 150 mM NaCl, 50 mM Tris-HCl, pH8.0, 10 mM MgCl_2_, 1 mM DTT and 0.1% NP-40 for at 37°C for 16 h. The reactions were then fractioned by sucrose gradient centrifugation. HBV capsids in each of viral DNA-containing fractions, as detected by a qPCR assay, were determined by a 1.5% native agarose gel electrophoresis-based particle gel assay. HBV DNA in the fractions were detected by Southern blot hybridization with a riboprobe that specifically hybridizes to negative strand of HBV DNA. The relaxed circular (RC) DNA, double-stranded linear (DSL) DNA and full-length single stranded (SS) DNA species were indicated. Sucrose concentration (w/v %) of each of the fractions is provided. The mature forms of HBV DNA species (RC and DSL) are highlighted in red-dash lined box.

Although results presented above indicate that CpAM treatment induces the exposure of rcDNA in nucleocapsids to DNase I digestion, the extent of disruption on the structure of mature nucleocapsids by the different CpAMs remains to be determined. We therefore examined whether the mature viral DNA species, rcDNA and double-stranded linear (dsl) DNA, were still associated with capsids after CpAM treatment. The assumption is that if CpAM treatment severely disrupts the mature nuclocapsids and results in releasing of viral DNA, we anticipate that rcDNA and dslDNA species will not co-sediment with any form of capsids. However, if CpAM treatment only mildly disrupts the mature nucleocapsids and causes the exposure of viral DNA that is still associated with capsid structure, we anticipate to see the rcDNA and/or dslDNA species will co-sediment with capsids. To this end, HBV capsids prepared from HepAD38 cells were mock treated or incubated with ENAN-34017, Bay 41–4109 or GLS4 in endogenous DNA polymerase reactions without dNTPs. The reactions were then fractionated by sucrose density gradient ultracentrifugation. The amounts of total capsids and capsid-associated DNA in each of the fractions were analyzed by a 1.5% native agarose gel electrophoresis-based particle gel assay [39]. As shown in the upper panels of Fig 8 A to 8D, compared to mock-treated control, treatment with any of the three CpAMs did not alter the sedimentation profile of capsids and capsid associated HBV DNA. These results are consistent with the observation that CpAMs only affect the mature nuclocapsids that constitute only a small portion of total capsids (Fig 7 and S7 Fig). However, analysis of viral DNA by Southern blot hybridization in each of the fractions revealed that rcDNA and dslDNA were lost or reduced from capsids sedimenting to 21% to 25% sucrose after Bay 41–4109 or GLS4 treatment, but the majority of rcDNA and dslDNA still co-sediment with capsids after ENAN-34017 treatment (Fig 8 A to 8D, lower panels). Due to the small amounts of rcDNA and dslDNA and low sensitivity of Southern blot hybridization assay, we were not able to identify the location or distribution of the disassociated DNA species in the gradients. Nevertheless, those results indicate that while Bay 41–4109 or GLS4 treatment most likely induced structural changes that were significant enough to cause loss of rcDNA and dslDNA from majority of the mature nucleocapsids, the less active compound ENAN-34017 might only induce structural changes that render the DNA content susceptible to DNase digestion. These two distinct degrees of conformational alteration may correspond to a subtle increase in “capsid breathing” for ENAN-34017, versus extensive rearrangement of the capsid subunit organization for BAY-41-4109 and GLS4 [41].

## Discussion

**Fig 9:**
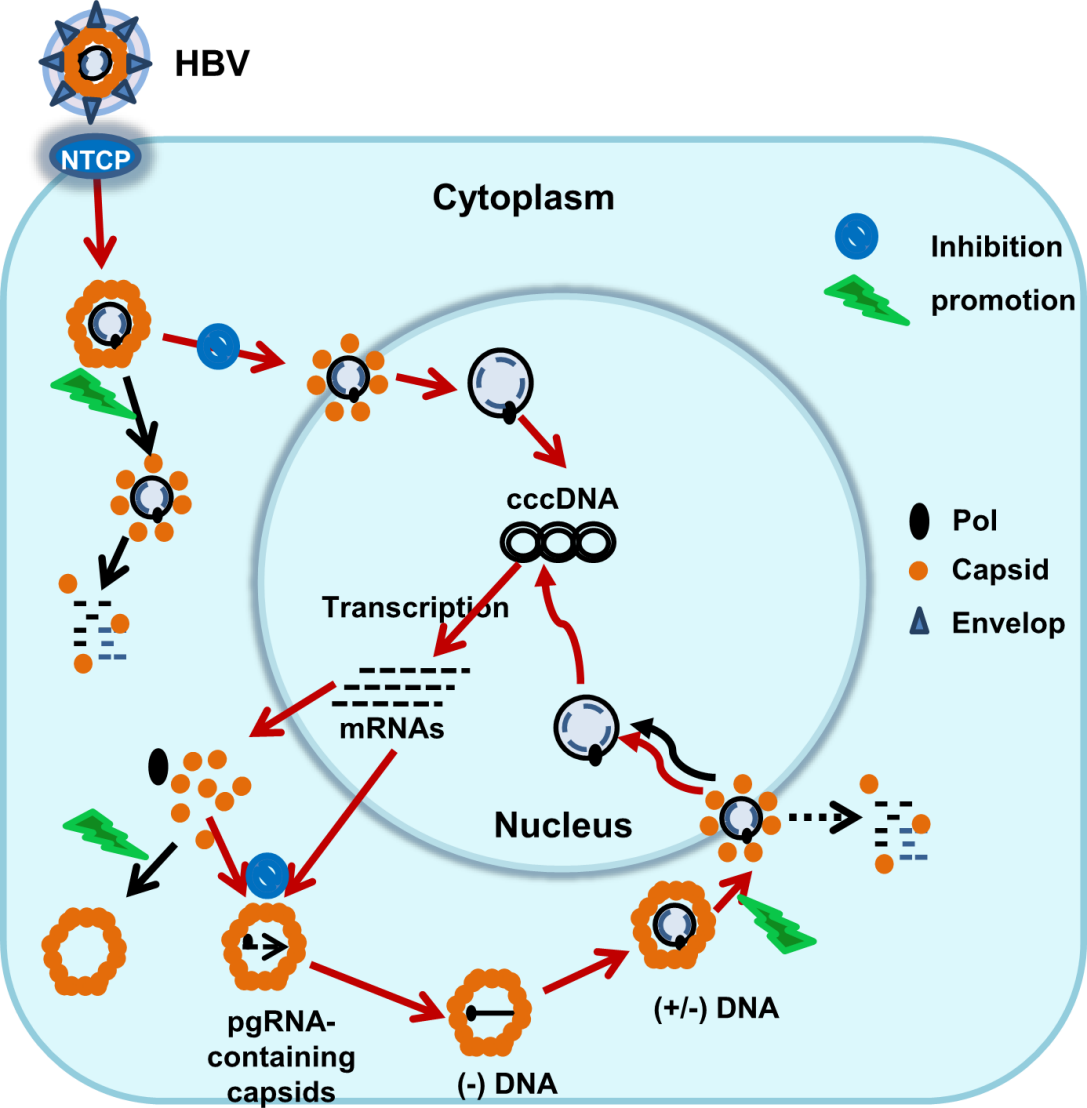
A schematic representation of the multiple mode of action of CpAMs on HBV capsid assembly and disassembly as well as their effects on cccDNA biosynthesis. The red arrowed lines indicate the normal processes in viral replication. The black arrowed lines indicate CpAMs-induced processes. See text for detailed explanation.

The genomes of all viruses are wrapped with capsid protein(s) to form nucleocapsids. Unlike viral enzymes that often have host cellular homologues, host cells do not encode proteins that are structurally and functionally similar to viral capsid proteins. Therefore, viral capsid proteins are ideal and highly selective antiviral targets [42]. In addition to serving as a vehicle for transmission of viral genomes between host cells, HBV nucleocapsids have other unique functions in the viral life cycle [4,9,27]. As illustrated in Fig 9, first, HBV DNA replication occurs exclusively within the cytoplasmic nucleocapsids, by reverse transcription of viral pgRNA first into negative-strand DNA and then double-stranded rcDNA. Second, unlike all other viruses where the progeny nucleocapsids have only one destination, *i*.*e*., to be secreted as virions, the HBV progeny nucleocapsids can also deliver their rcDNA into the nuclei to synthesize cccDNA. Due to superinfection exclusion [43,44], it is reasonable to consider that the activity of the intracellular amplification pathway is the key determinant of cccDNA pool size in infected hepatocytes [45]. Due to the large amounts of progeny nucleocapsids in the cytoplasm, this intracellular cccDNA amplification pathway must be, and actually is, tightly controlled by viral and host factors [7,34,37,46]. Finally, the exclusive replication in nucleocapsids and the spatially-controlled disassembly and delivery of rcDNA into nuclei at nuclear pore complexes protect the viral DNA from recognition by cytoplasmic DNA sensors, and thus favor the persistent infection by HBV [8,9,47]. Hence, it has been speculated that targeting HBV core protein may disrupt multiple steps of HBV replication and activate innate immune response in virally infected cells. However, as mentioned, while several distinct chemotypes of HBV CpAMs have been shown to disrupt viral nucleocapsid assembly and consequentially inhibit viral genome replication, their effects on other aspects of nucleocapsid function have not been investigated. Herein, we provide evidence suggesting that selected HAPs and SBAs are able to interact with nucleocapsids from virions and mature forms of rcDNA-containing nucleocapsids in the cytoplasm and subsequently interfere with their function of delivering viral rcDNA to the nuclei for cccDNA synthesis.

Although the structural basis for the CpAMs to target the highly selected sub-populations of nucleocapsids remains to be determined, our results are consistent with previous findings that mature HBV nucleocapsids are intrinsically unstable or fragile [48], probably due to the stiffness of rc and dslDNA, which imposes bending energy and electrostatic repulsion to the capsid shell [49]. Moreover, viral DNA synthesis in the nucleocapsids assembled from mutant DHBV core proteins with a serial N-terminal inertions destabilized mutant nucleocapsids, rendering mature viral DNA selectively sensitive to nuclease digestion [50]. Therefore, it is possible that the intrinsic instability of mature nucleocapsids, due to the completion of double-stranded DNA, confers the selective sensitivity for CpAMs to induce disassembly. Considering the result that CpAM treatment induces the accessibility of all forms of virion DNA, either rcDNA or partially double-stranded DNA, to DNase I digestion, a possible explanation is that like many other viruses, structural shifts or maturation of nucleocapsids may occur during or after virion assembly and secretion, and confer susceptibility to CpAMs [51]. Moreover, a recent report showed that the carboxyl-terminal domain of core proteins is hypophosphorylated in DNA-containing virions, but remains hyperphosphorylated in intracellular DNA-containing nucleocapsids [52]. Although the C-terminal arginine-rich domain of HBV core protein is not directly involved in the binding of CpAMs [39], the dephosphorylation may alter the interaction between core protein and viral DNA [53], and thus affect the nucleocapsid structure in a manner distinct from that of the hyperphosphorylated state [54]. In addition, the sensitivity of partially double-stranded DNA-containing cytoplasmic nucleocapsids to the increased concentrations of CpAMs strongly suggests that elongation of positive-stranded DNA toward rcDNA induces incremental, or gradual, structural changes favoring specific interaction with CpAMs. Structural biology studies with purified double stranded DNA-containing capsids may reveal those important structure differences. However, it is technically challenging to purify sufficient amounts rcDNA-containing HBV capsids for Cryo-EM analyses.

Although our study revealed correlations between altered cccDNA synthesis and induction of nucleocapsid destablization by CpAMs, the molecular mechanisms on how the CpAM-induced nucleocapsid structural changes disrupt cccDNA formation in *de novo* infection, but accelerate cccDNA synthesis from progeny intracellular nucleocapsids remain to be determined. However, reasonable speculations can be made based on current knowledge. As illustrated in Fig 9, on one hand, in *de novo* infection, CpAMs may interact with nucleocapsids in virions during endocytic entry or immediately after their release into the cytoplasm to induce viral DNA exposure or release from nucleocapsids. The premature disassembly of nucleocapsids results in viral DNA release and decay by cytoplasmic DNases before its arrival to the nuclear pore complex for nuclear import and subsequent cccDNA synthesis. On the other hand, due to the shorter distance traveled as compared to the *de novo* infection, the intracellular progeny rcDNA-containing nucleocapsids may reach nuclear pore complexes before a certain stage of their disassembly and CpAM-induced uncoating actually accelerates the release of rcDNA into the nuclei and thus cccDNA formation. Of course, a fraction of rcDNA decay in the cytoplasm may also occur. In fact, this interpretation is in agreement with recent findings that enhanced destabilization of mature rcDNA containing nucleocapsids, due to unidentified host cellular factors in mouse hepatocytes or a single amino acid substitution (I126A) in core protein, significantly reduced the accumulation of rcDNA, but increased the DP-rcDNA and cccDNA synthesis [7,37].

It had not escaped our attention that the released or exposed rc/dslDNA in the cytoplasm may activate innate DNA sensors [55–58]. However, cytokine response was not detected in CpAM-treated cells. Whether this is due to the deficiency of cyclic GMP-AMP synthase (cGAS)- stimulator of interferon genes (STING) pathway in hepatocytes and hepatoma cells [59–61] or efficient digestion of the released viral DNA by cytoplasmic nucleases [62] is currently under investigation.

While inhibition of cccDNA synthesis from *de novo* infection is obviously a beneficial therapeutic effect, the acceleration of intracellular cccDNA amplification is detrimental. However, the potent inhibition of pgRNA encapsidation by CpAMs will quickly block viral DNA replication and production of mature progeny nucleocapsids and consequential cccDNA synthesis. Therefore, the only chance for CpAMs to accelerate cccDNA synthesis during antiviral therapy is at the initial period of treatment to promote cccDNA synthesis from pre-existing mature nucleocapsids in HBV infected cells. Fortunately, treatment of HepAD38 cells supporting steady-state HBV DNA replication with ETV and CpAMs to mimic the initial stage of antiviral treatment *in vivo* did not observe a significant increase of cccDNA accumulation within first 24 h of treatment. Instead, similar to ETV, CpAMs prevented amplification of cccDNA pool during a prolonged treatment, as compared with mock-treated control (S9 Fig). In fact, those results are consistent with the observation that CpAM treatment of primary human hepatocytes after establishment of HBV infection did not alter the amount of cccDNA [30]. Taking together, the results imply that CpAMs only significantly increase the amount of cccDNA by acceleration of ongoing cccDNA synthesis, such as under the condition of fast cccDNA synthesis after release from PFA arrested HBV DNA replication, but cannot accelerate the very low (or negligible) rate of cccDNA synthesis to significantly increase the amount of cccDNA in cells with established HBV infection. Mechanistically, as stated above, it is possible that only when CpAM-induced disassembly of mature nucleocapsids occurs at or near the nuclear pore complex may facilitate the import of rcDNA into the nuclus for cccDNA synthesis, but the mature nucleocapsids at such status may not exist in a significant amount in hepatocytes with established infection. Nevertheless, careful monitoring of cccDNA synthesis during the early phase of CpAM treatment *in vivo* in animals and in clinical trials and pretreatment or combination with viral DNA polymerase inhibitors might be considered.

## Materials and methods

### Cell cultures, drugs and antibodies

The HepAD38 cell line (obtained from Dr. Christoph Seeger at Fox Chase Cancer Center, Philadelphia) supporting HBV pgRNA transcription and subsequent viral DNA replication in a tetracycline (tet)-inducible manner was maintained as previously described [63]. C3A cell line [a derivative of HepG2 (ATCC HB-8065)] (ATCC CRL-10741) was maintained in DMEM/F-12 (1:1) medium supplemented with 10% FBS (GEMINI Bio-Products), 100 U/ml penicillin, 100 μg/ml streptomycin. Entecavir (ETV) is a gift from Dr. William S. Mason at Fox Chase Cancer Center, Philadelphia [64]. Foscarnet was purchased from Sigma. Capsid assembly modulators ENAN-34017 and Bay41-4109, GLS4 and AT-61 were described previously [35,65]. Myrcludex B is a gift of Dr. Stephan Urban at Heidelberg University, Germany [26]. Alpha-interferon (IFN-α) was purchased from PBL Assay Science. Rabbit anti-HBc antibody was obtained from Dako (B0586).

### Establishment of NTCP-expressing C3A cell line and HBV infection

Human sodium taurocholate cotransporting polypeptide (NTCP) gene coding sequence was amplified from a cDNA clone purchased from Origene (SC118232). A carboxyl-terminal C9 tag was added by PCR amplification with the primers harboring C9 tag sequence and NotI & Bam HI restriction enzyme sites. The purified PCR fragments were digested with restriction enzymes Not I and Bam HI and subsequently cloned into a pQCXIP vector (Clontech). VSV G protein pseudotyped retroviruses were packaged in GP2-293 cells as previously described [66]. C3A^hNTCP^ cell line stably expressing human NTCP was established by infection of C3A cell line with the pseudotyped retroviruses and selected with medium containing 2 μg/ml of puromycin. Puromycin-resistant cells were expanded into cell line and designated as C3A^hNTCP^. Proper expression of NTCP was confirmed by immunofluorescence and Western blot assays.

C3A^hNTCP^ cells were seeded into collagen-coated 24-well plates at a density of 4×10^5^ cells per well and cultured in complete DMEM medium containing 3% dimethyl sulfoxide (DMSO). One day later, the cells were infected with HBV prepared from HepAD38 cell culture media at a MOI of 500 genome equivalents per cell in DMEM containing 4% PEG-8000. The inoculums were removed at 24 h post infection and the cultures were maintained in complete DMEM medium containing 3% DMSO until harvesting.

### Extraction and analyses of HBV DNA and RNA by hybridization and real-time PCR assays

HBV core DNA and RNA extraction from infected C3A^hNTCP^ or HepAD38 cells, Southern blot hybridization, and real-time PCR analyses were performed as described previously [35,65]. HBV cccDNA from HBV-infected C3A^hNTCP^ cells and HepAD38 cells were extracted by a modified Hirt DNA extraction procedure [33]. A fraction of Hirt DNA preparation was digested with 1 unit of plasmid-safe adenosine triphosphate (ATP)-dependent deoxyribonuclease (PSAD) (Epicentre Technologies) in a 25 μL reaction for 1 h at 37°C to remove rcDNA. The DNase was inactivated by incubation of the reactions for 30 min at 70°C. cccDNA in the PSAD-treated samples were quantified by a real time PCR assay with primer sequences GGGGCGCACCTCTCTTTA (forward) and CCACCCAGGTAGCTAGAGTCATTAG (reverse). The real-time PCR was performed using the SYBR Premix Ex Taq on a LightCycler 480 II (Roche) as the following reaction procedure: 95°C for 10 min then 45 cycles of 95°C for 30 s, 60°C for 5 s, and 72°C for 30 s. The amount of HBV cccDNA in a DNA preparation was determined by real-time PCR using a plasmid containing HBV genotype D genome as the standard.

For Southern blot hybridization, the Hirt DNA samples were denatured at 88°C for 5 minutes and chilled in ice. This procedure completely denatures deproteinized rc-DNA (DP-rcDNA) into single stranded DNA, whereas cccDNA will remain as double stranded circular DNA [67]. The denatured Hirt DNA samples without or with further digestion with restriction enzyme Eco RI to linearize cccDNA into double-stranded linear DNA were resolved in 1.5% agarose gel and transferred onto Hybond-XL membrane. The membrane was probed with α-^32^P-UTP labeled minus strand specific full-length HBV riboprobe.

#### HDV production, infection and RNA quantification

Cell culture derived HDV particles were generated as described previously [68]. Briefly, Huh7 cells (obtained from Dr. Christoph Seeger at Fox Chase Cancer Center, Philadelphia) were co-transfected with a plasmid containing three copies of HDV cDNA sequence (pSVLD3) and a plasmid expressing HBV envelope proteins L, M and S (pSV45H) [69]. HDV particles were harvested from the media between 6 to 9 and 9 to 12 days post transfection. After clarification by centrifugation, particles were precipitated in the presence of 10% PEG-8000 and the pellet was dissolved in TAN buffer (50 mM Tris-HCl, pH 8.0 and 100 mM NaCl) at 1% volume of the original culture medium. For infection, C3A^hNTCP^ cells were seeded into collagen-coated 24-well plates at a density of 4×10^5^ cells per well and cultured in complete DMEM medium containing 3% dimethyl sulfoxide (DMSO). One day later, the cells were infected with HDV at a MOI of 500 genome equivalents per cell in DMEM containing 4% PEG-8000. The inoculums were removed at 24 h post infection and the cultures were maintained in complete DMEM medium containing 3% DMSO until harvesting. Intracellular HDV genomic and antigenomic RNAs were quantified by qRT-PCR using strand-specific primers as described previously [68].

#### Purification of HBV virions and cytoplasmic capsids

HBV virions were prepared from a HBV carrier’s serum (from Bioreclamation IVT, The Complete Resources for All Biologicals) by sucrose gradient centrifugation. Briefly, 1 ml of human sera was layered onto a 2-ml 20% (wt/vol) sucrose cushion in 0.15 M NaCl, 0.02 M Tris-HCl, pH 7.4, and centrifuged for 3 h at 45,000 rpm in SW55 rotor at 4°C. The supernatant fluid was removed and pellet was dissolved in TNE buffer (0.15 M NaCl; 0.01 M Tris-HCl, pH 7.4; and 0.1 mM EDTA). To prepare intracellular HBV capsids, HepAD38 cells were cultured in tetracycline-free medium with or without 2 mM Foscarnet (PFA) for 6 days with daily medium change. The cells were then lysed with chilled lysis buffer containing 10 mM Tris-HCl (pH 8.0), 1 mM EDTA, 0.1% Nonidet P-40 and 8% sucrose on ice for 10 minutes, the lysate was centrifuged at 10,000 g for 5 min to remove the nuclei and cell debris. The clarified supernatant was overlaid onto a 10–55% (w/w) sucrose gradient and centrifuged at 24,000 rpm for 16 h at 4°C using a Beckman SW28 rotor. Nineteen 2-ml fractions were collected from the bottom of the cushion. Ten microliters of each fraction was dot-blotted onto the nitrocellulose membrane and HBV core protein was detected by incubation with a rabbit antibody against HBV core protein (DAKO, B0586). The bound antibody was detected by IRDye secondary antibodies and visualized by Li-COR Odyssey system. HBV core protein-positive fractions were pooled together and the sucrose concentration was adjusted to 10% by addition of TNE buffer. The diluted sample was overlaid on a 20% sucrose cushion and centrifuged at 45,000 rpm for 3 h at 4°C using Beckman SW55 rotor. The pellet was resuspended in TNE buffer.

#### Endogenous DNA polymerase reaction

An endogenous DNA polymerase reaction (EPR) mixture was assembled with 20 μl of HBV virion or capsid preparation, 25 μl of 2x EPR buffer which consisted of 0.15 M NaCl, 0.1 M Tris-HCl (pH 8.0), 20 mM MgCl2, 2 mM dithiothreitol, 0.2% (vol/vol) Nonidet P-40. dNTP and capsid assembly modulators were added, as indicated, to 0.1 mM or a specified final concentration, respectively. Water was added to bring the reaction volume to 50 μl. After incubation at 37°C for an indicated period of time, the reaction was subjected for extraction of viral DNA with or without prior DNase I digestion at 37°C for 30 min. The viral DNA were resolved in 1.5% agarose gel and transferred onto Hybond-XL membrane. The membrane was probed with α-^32^P-UTP labeled minus strand specific full-length HBV riboprobe.

#### Particle gel assay

The cytoplasmic HBV capsids were analyzed by a native agarose gel electrophoresis-based assay. Briefly, ten microliters of endogenous DNA polymerase reaction or fraction of sucrose gradient centrifugation were resolved by electrophoresis through a 1.5% agarose gel and transferred to a nitrocellulose membrane by blotting with TNE buffer (10 mM Tris-HCl, pH 7.5, 150 mM NaCl, and 1 mM EDTA). HBV capsids on the membrane were detected with a procedure described previously [65].

#### Ultracentrifugation analysis of HBV capsid integrity

Two hundred microliters of the purified HBV capsids were mixed with 250 μl of reaction buffer containing 0.15 M NaCl, 0.1 M Tris-HCl (pH 8.0), 20 mM MgCl_2_, 2 mM dithiothreitol, 0.2% (vol/vol) Nonidet P-40. Capsid assembly modulators were added as indicated and water was provided to bring the reaction volume to 500 μl. After incubation at 37°C for the indicated period of time, the reaction was loaded onto a 15% to 30% linear sucrose gradient in TNE buffer and spun at 27,000 rpm for 4 hr (Beckman, Rotor SW28). 1.5mL fractions were collected from the bottom of the centrifugation tube by blood collection set. Sucrose concentration of each fraction was measured by Refractor (Mettler Toledo). The rest of each fraction was mixed with 3mL TNE buffer and pelleted by centrifugation at 46,000 rpm for 3 hr. Pellet was dissolved in 40 μL TNE buffer. 20μL of capsid solution were fractionated in a 1.5% agarose gel electrophoresis and transferred to a nitrocellulose membrane to detect HBV capsid by probing with anti-HBcAg antibody. Viral DNA was extracted from the remaining sample of reactions and detected by Southern blot hybridization with α-^32^P-UTP-labeled minus strand-specific full-length HBV riboprobe.

## Supporting information

S1 Fig Chemical structures of representative CpAMs used in this study. (TIF)

S2 Fig Kinetic analysis of HBV infection of C3A^hNTCP^ cells. C3A^hNTCP^ cells were mock-infected or infected with HBV at a MOI of 500 genome equivalents and cells were harvested at the indicated days post infection. HBV cccDNA **(A)**, pgRNA **(B)** and cytoplasmic core DNA **(C)** were quantified by real-time PCR assays. HBsAg in culture medium was measured by using an ELISA assay kit (Autobio) **(D)**. **(E)** The infected cells were fixed at 3 and 8 days post infection (dpi) and HBcAg was detected by indirect immunofluorescent staining (red). The cell nuclei were stained with DAPI (blue). The images were captured with a Nikon X71 microscopy. **(F)** Detection of HBV replication intermediates by hybridization assays. *Top panel*, Hirt DNA extracted from the cells was denatured at 88°C for 5 min to denature DP-rcDNA into single stranded DNA and followed by restriction with EcoRI to convert cccDNA into unit-length dslDNA (labeled as CCC/EcoRI) and detected by Southern blot hybridization. Unit-length HBV linear DNA served as a molecular weight marker. *Middle panel*, HBV pgRNA and mRNAs specifying envelope proteins (envRNAs) were detected by Northern blot hybridization. 28S and 18S ribosomal RNA (rRNA) served as loading controls. *Lower panel*, HBV core DNA were detected by Southern blot hybridization. The relaxed circular (RC) DNA, double-stranded linear (DSL) DNA and full-length single stranded (SS) DNA species were indicated.
(TIF)

S3 Fig HBV CpAMs, but not DNA polymerase inhibitor (entecavir, ETV) and IFN-α, inhibit cccDNA formation from *de novo* infection. **(A)** C3A^hNTCP^ cells were infected with HBV at an MOI of 500 genome equivalents. The cells were treated and harvested as the depicted schedule. **(B)** Hirt DNA were extracted and HBV DP-rcDNA were digested by treatment with PSAD. HBV cccDNA were quantified by a real-time PCR assay and expressed and copies per 4*10^5^ cells. Concentrations of the drugs used in the experiment were 1 μM of ETV, 2.5 μM of Bay 41–4109, 5 μM of ENAN-34017, 1 μM of GLS4 and 1,000 IU/ml of IFN-α. Differences in viral cccDNA between mock-treated control and treated cultures in each time point were statistically analyzed (t-test, * *p* <0.05, ** *p* <0.01, *** *p* <0.001).
(TIF)

S4 Fig HBV capsid assembly modulators do not inhibit HDV infection of C3A^hNTCP^ cells. **(A)** C3A^hNTCP^ cells were infected with HDV at an MOI of 500 genome equivalents. The cells were treated and harvested as the schedule depicted. **(B)** The genomic and anti-genomic HDV RNA were quantified by real-time RT-PCR assays. Concentrations of the drugs used in the experiment are 100 nM of MyrB, 1 μM of ETV, 2.5 μM of Bay 41–4109, 5 μM of ENAN-34017 and 1,000 IU/ml of IFN-α.
(TIF)

S5 Fig Detection and identification of cccDNA in HepAD38 cells by Southern blot assay. Hirt DNA preparations made from HepAD38 cells cultured in the absence of tetracycline for 8 days were separated on an agarose gel without treatment (lane 2), after denaturalization at 88°C for 5 min (lane 3) and digestion with EcoRI after denaturalization at 88°C for 5 min (lane 4). HBV DNA species were detected by Southern blot hybridization and denoted.
(TIF)

S6 Fig HBV capsid assembly modulators do not inhibit cccDNA formation through intracellular amplification pathway. **(A)** HepAD38 cells were sequentially treated with 2 mM of PFA and 1 μM of ETV, 2.5 μM of Bay 41–4109, 1 μM of GLS4 or 5 μM of ENAN-34017 and harvested as the schedule depicted. The cytoplasmic core DNA (**B**), cccDNA and DP-rcDNA (**C**) were analyzed Southern blot hybridization. The relaxed circular (RC) DNA, double-stranded linear (DSL) DNA and full-length single stranded (SS) DNA species are indicated. For cccDNA and DP-rcDNA analysis, before loading, Hirt DNA were denatured at 88°C for 5 min and followed by digestion with EcoRI.
(TIF)

S7 Fig HBV capsid assembly modulators dose-dependently induce disassembly of mature nucleocapsids. HBV capsids prepared from HepAD38 cells were incubated with the indicated concentrations of Bay 41–4109 **(A)**, GLS4 **(B)** or ENAN-34017 **(C)** in reactions containing 150 mM NaCl, 50 mM Tris-HCl, pH8.0, 10 mM MgCl_2_, 1 mM DTT and 0.1% NP-40 at 37°C for 16 h and then left untreated or treated with 10 μg/ml DNase I for 30 mins. Viral DNA was extracted and resolved by agarose gel electrophoresis and detected by Southern blot hybridization with full-length riboprobe recognizing minus strand DNA. The relaxed circular (RC) DNA, double-stranded linear (DSL) DNA and full-length single stranded (SS) DNA species are indicated.
(TIF)

S8 Fig GLS4 induces mature nucleocapsid disassembly in HepAD38 cells. HepAD38 cells were cultured in tet-free medium for six days and then mock-treated or treated with 1μM GLS4 for the indicated period of time. The cells were harvested and cytoplasmic DNA was extracted without or with prior treatment of cell lysates with 10 μg/ml of DNase I for 30 mins. The viral DNA were resolved in agarose gel and detected by Southern blot hybridization with full-length riboprobe recognizing minus strand DNA. The relaxed circular (RC) DNA, double-stranded linear (DSL) DNA and full-length single stranded (SS) DNA species are indicated.
(TIF)

S9 Fig Effects of CpAMs on cccDNA synthesis in hepatoma cells with established HBV replication. (**A**) HepAD38 cells were cultured in the absence of tetracycline for 7 days to allow HBV pgRNA transcription, DNA replication and cccDNA synthesis to occur. The cells were then mock-treated or treated with ETV (1 μM), GLS4 (1 μM) or ENAN-34017 (5 μM). Cells were harvested on day 1, 2, 4 of treatment. Viral core DNA (**B**) and Hirt DNA (**C**) were extracted and analyzed Southern blot hybridization with a riboprobe specific to negative strand DNA. The relaxed circular (RC) DNA, double-stranded linear (DSL) DNA and full-length single stranded (SS) DNA species are indicated. For cccDNA and DP-rcDNA analysis, before loading, Hirt DNA were denatured at 88°C for 5 min and followed by digestion with Eco RI. 3.2 kb and 2.0 kb HBV DNA served as a molecular weight markers.
(TIF)
