# Supplementary figures and images for: HBV core protein allosteric modulators differentially alter cccDNA biosynthesis from de novo infection and intracellular amplification pathways
Source: PLoS Pathog. 2017 Sep 25;13(9):e1006658. doi: 10.1371/journal.ppat.1006658 (PMC5629035; doi:10.1371/journal.ppat.1006658)

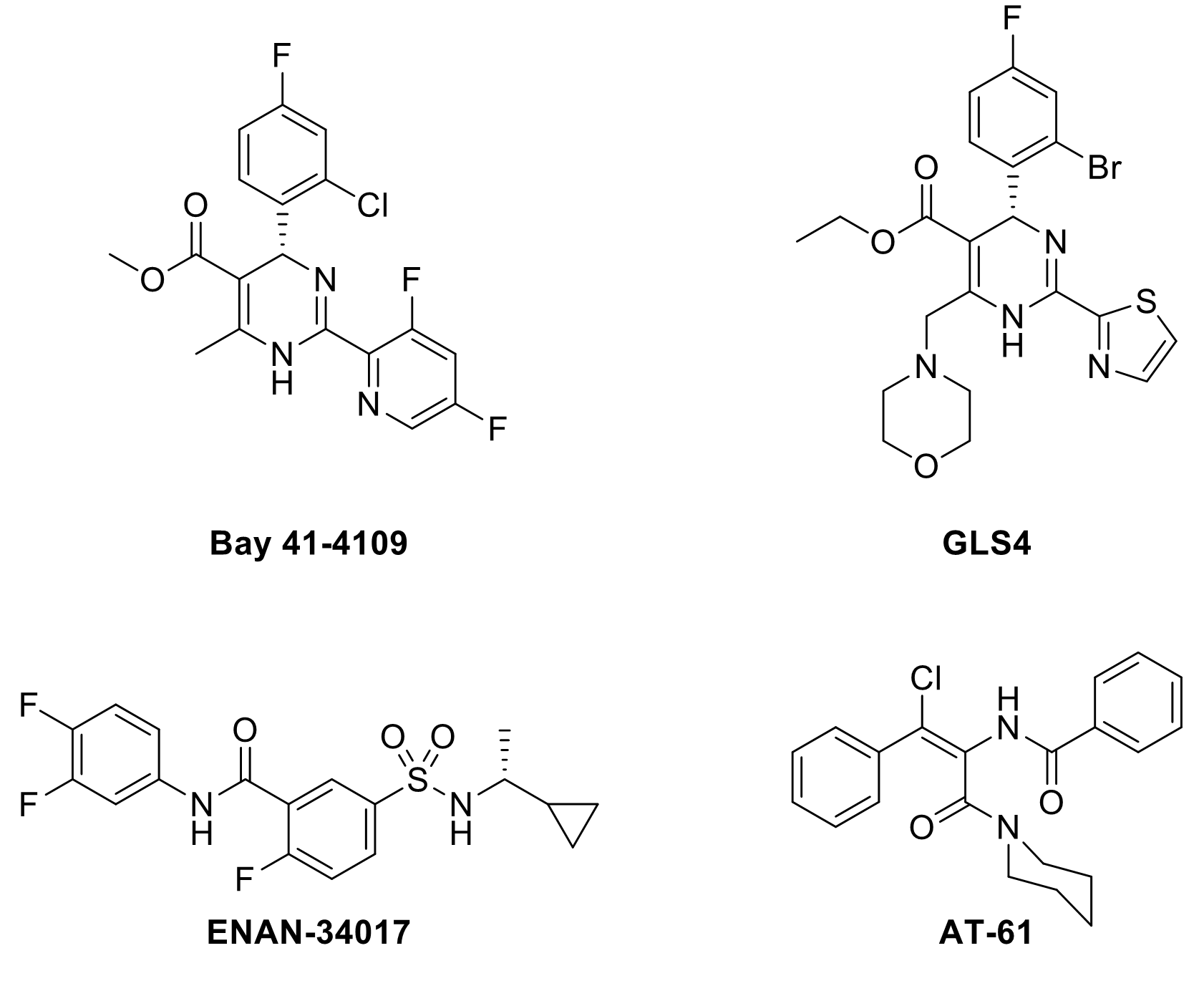

Supplement: S1 Fig — (TIF) [file ppat.1006658.s001.tif]

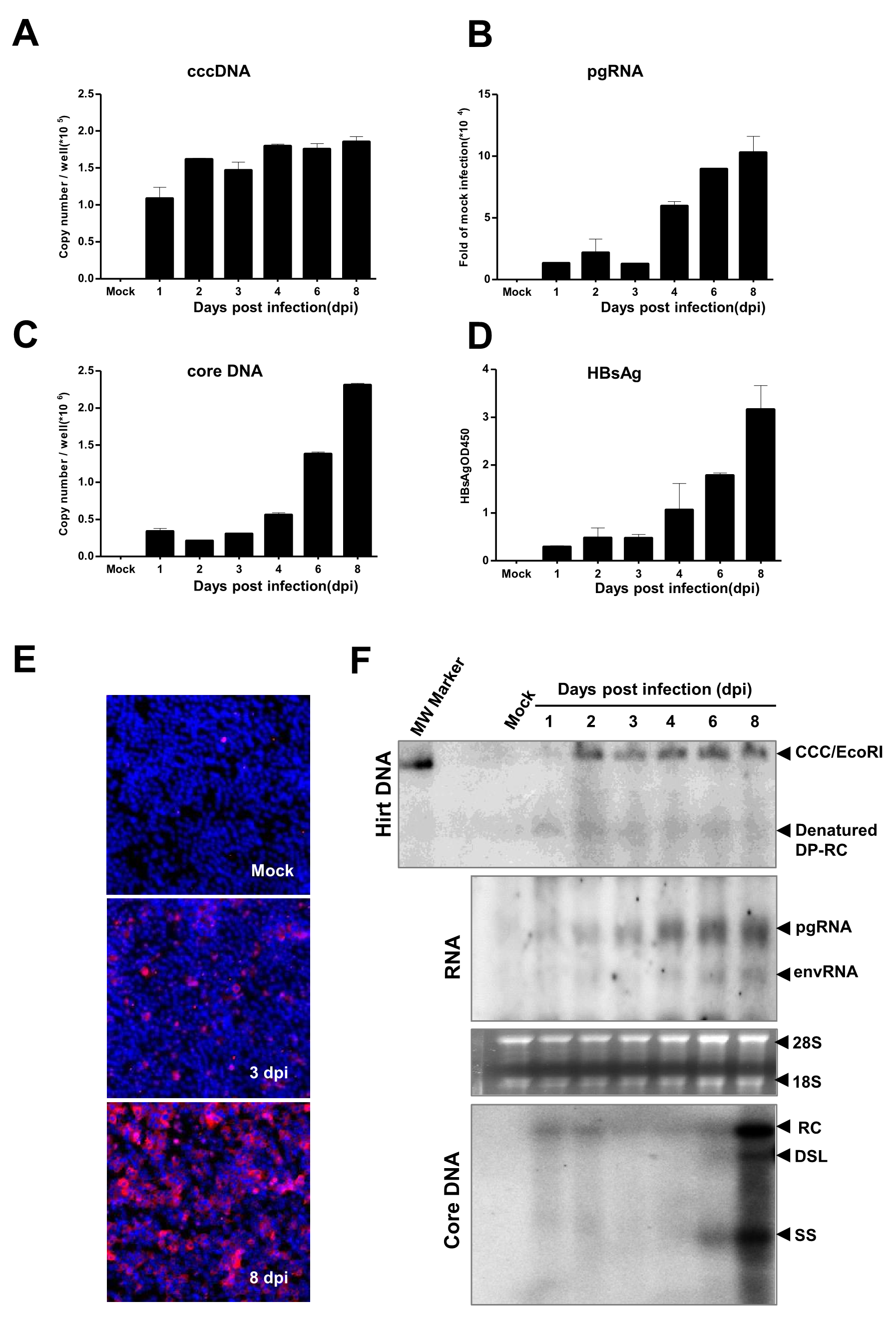

Supplement: S2 Fig — C3AhNTCP cells were mock-infected or infected with HBV at a MOI of 500 genome equivalents and cells were harvested at the indicated days post infection. HBV cccDNA (A), pgRNA (B) and cytoplasmic core DNA (C) were quantified by real-time PCR assays. HBsAg in culture medium was measured by using an ELISA assay kit (Autobio) (D). (E) The infected cells were fixed at 3 and 8 days post infection (dpi) and HBcAg was detected by indirect immunofluorescent staining (red). The cell nuclei were stained with DAPI (blue). The images were captured with a Nikon X71 microscopy. (F) Detection of HBV replication intermediates by hybridization assays. Top panel, Hirt DNA extracted from the cells was denatured at 88°C for 5 min to denature DP-rcDNA into single stranded DNA and followed by restriction with EcoRI to convert cccDNA into unit-length dslDNA (labeled as CCC/EcoRI) and detected by Southern blot hybridization. Unit-length HBV linear DNA served as a molecular weight marker. Middle panel, HBV pgRNA and mRNAs specifying envelope proteins (envRNAs) were detected by Northern blot hybridization. 28S and 18S ribosomal RNA (rRNA) served as loading controls. Lower panel, HBV core DNA were detected by Southern blot hybridization. The relaxed circular (RC) DNA, double-stranded linear (DSL) DNA and full-length single stranded (SS) DNA species were indicated. (TIF) [file ppat.1006658.s002.tif]

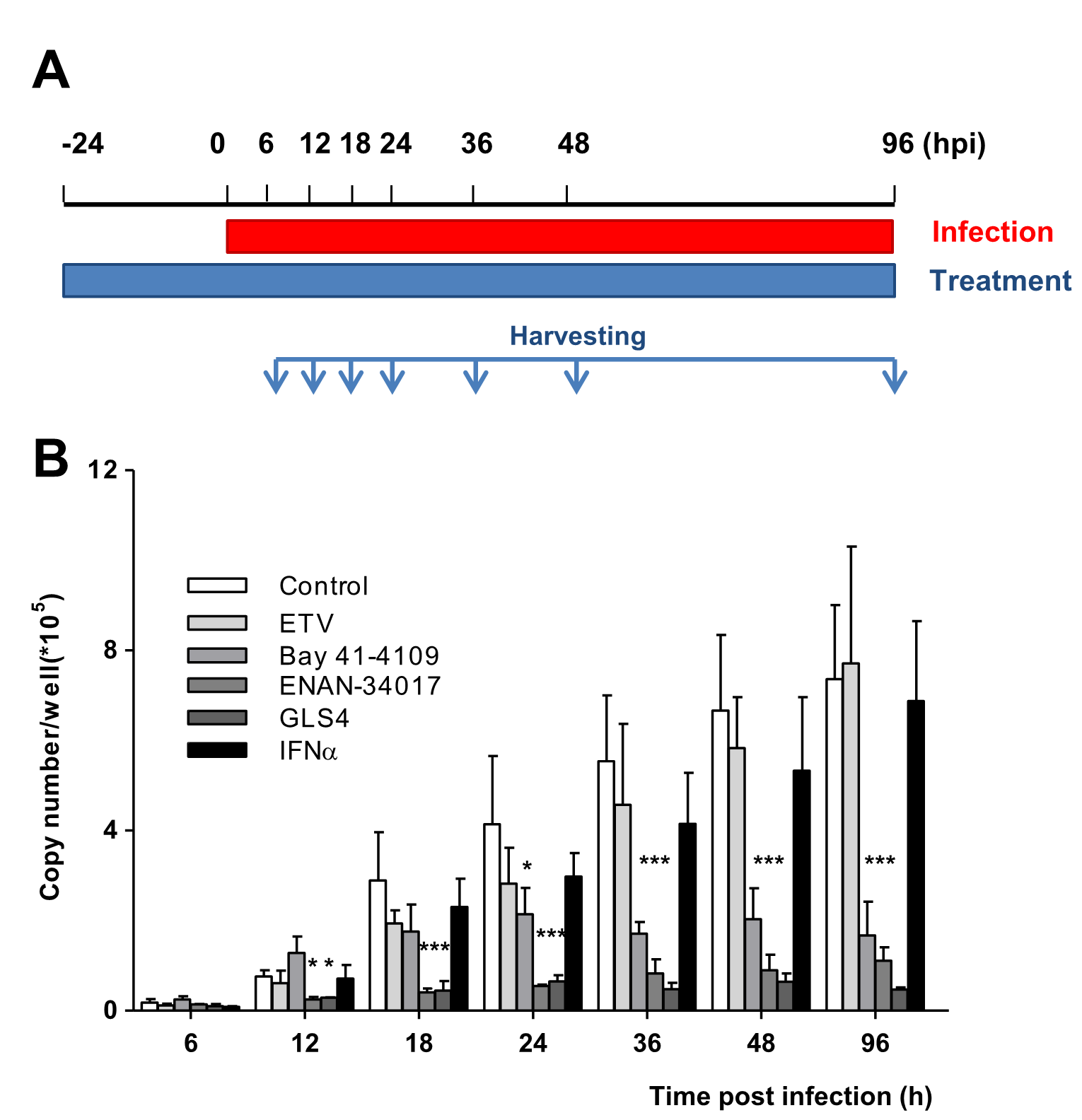

Supplement: S3 Fig — (A) C3AhNTCP cells were infected with HBV at an MOI of 500 genome equivalents. The cells were treated and harvested as the depicted schedule. (B) Hirt DNA were extracted and HBV DP-rcDNA were digested by treatment with PSAD. HBV cccDNA were quantified by a real-time PCR assay and expressed and copies per 4*105 cells. Concentrations of the drugs used in the experiment were 1 μM of ETV, 2.5 μM of Bay 41–4109, 5 μM of ENAN-34017, 1 μM of GLS4 and 1,000 IU/ml of IFN-α. Differences in viral cccDNA between mock-treated control and treated cultures in each time point were statistically analyzed (t-test, * p <0.05, ** p <0.01, *** p <0.001). (TIF) [file ppat.1006658.s003.tif]

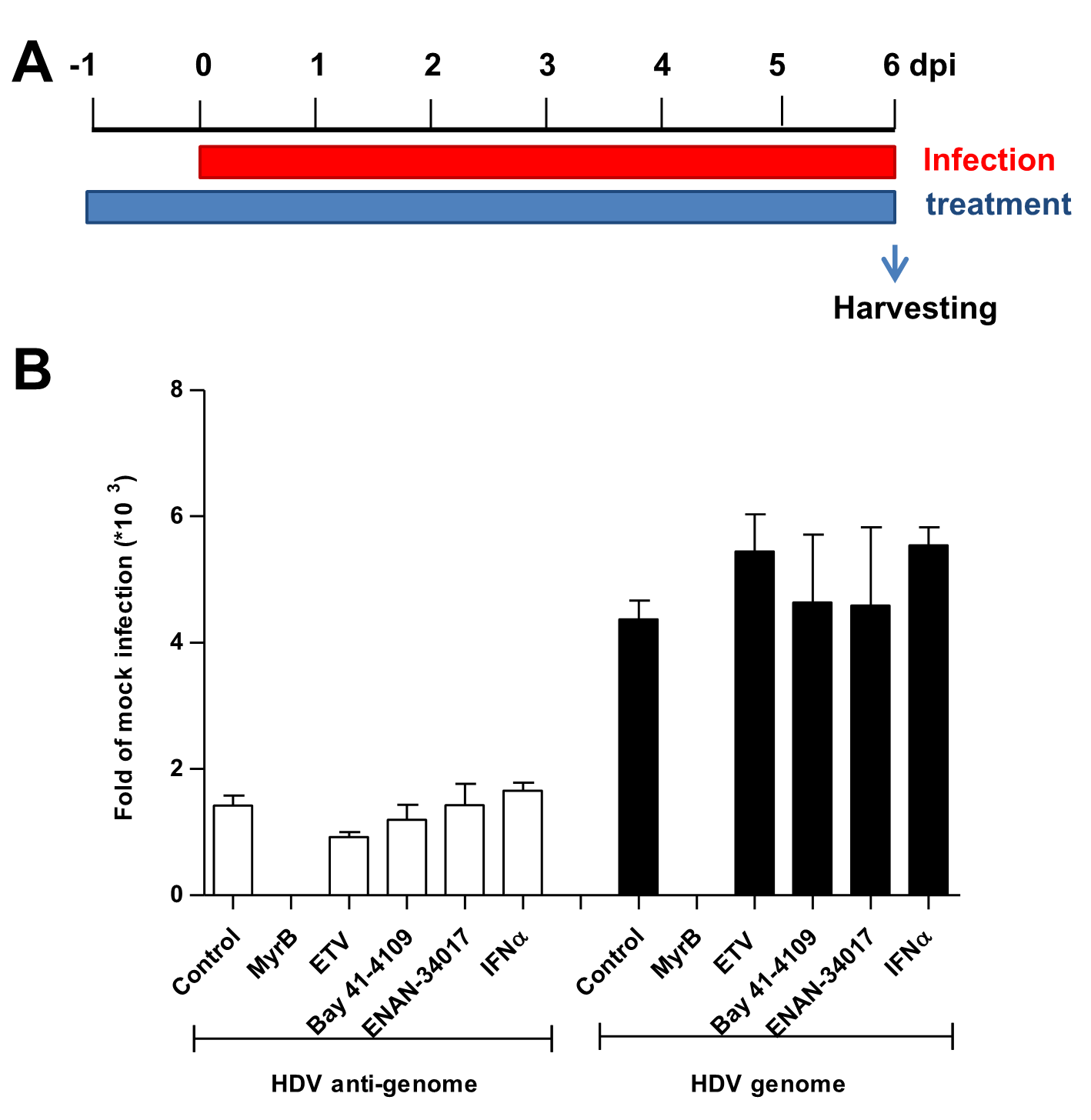

Supplement: S4 Fig — (A) C3AhNTCP cells were infected with HDV at an MOI of 500 genome equivalents. The cells were treated and harvested as the schedule depicted. (B) The genomic and anti-genomic HDV RNA were quantified by real-time RT-PCR assays. Concentrations of the drugs used in the experiment are 100 nM of MyrB, 1 μM of ETV, 2.5 μM of Bay 41–4109, 5 μM of ENAN-34017 and 1,000 IU/ml of IFN-α. (TIF) [file ppat.1006658.s004.tif]

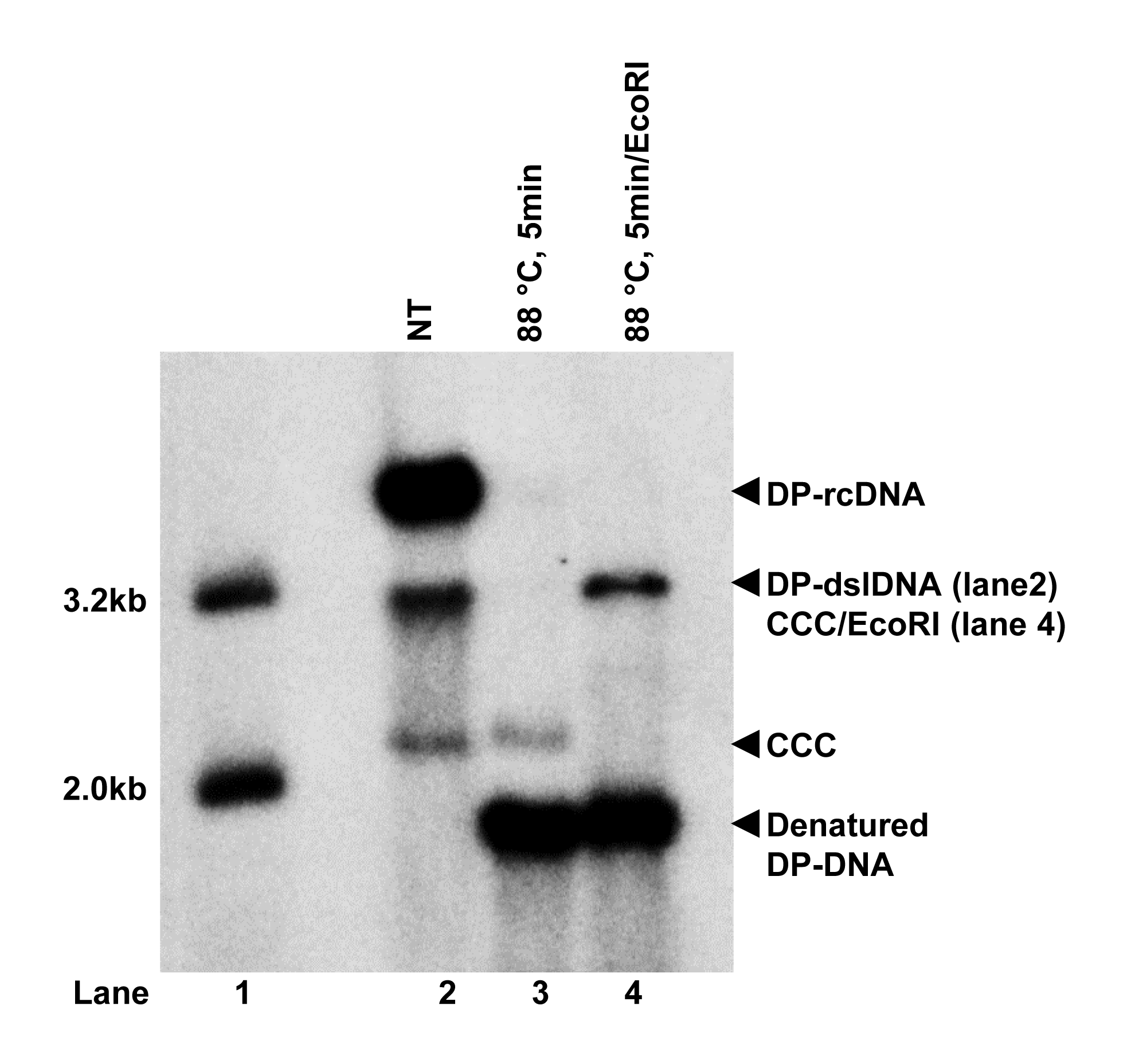

Supplement: S5 Fig — Hirt DNA preparations made from HepAD38 cells cultured in the absence of tetracycline for 8 days were separated on an agarose gel without treatment (lane 2), after denaturalization at 88°C for 5 min (lane 3) and digestion with EcoRI after denaturalization at 88°C for 5 min (lane 4). HBV DNA species were detected by Southern blot hybridization and denoted. (TIF) [file ppat.1006658.s005.tif]

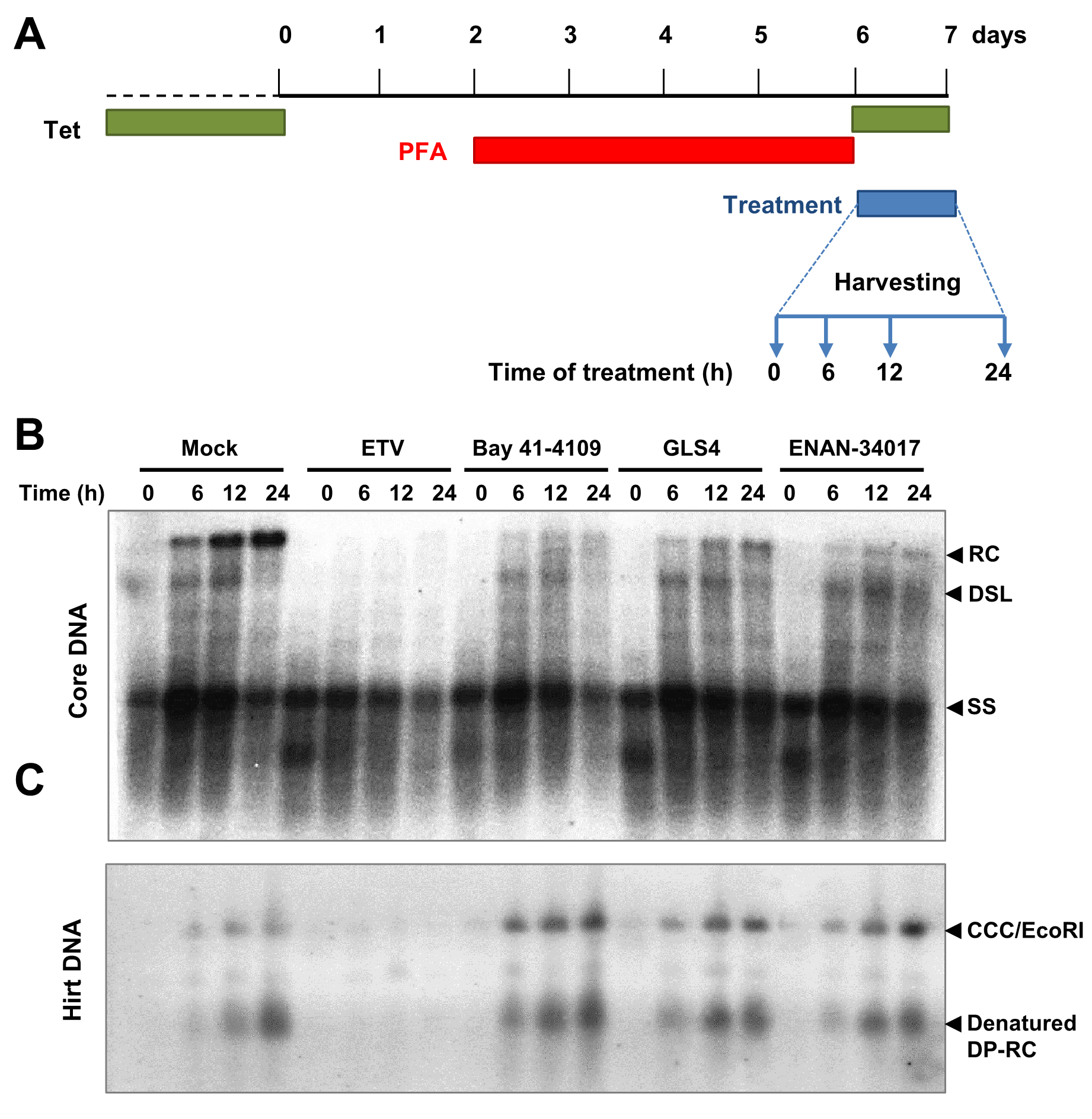

Supplement: S6 Fig — (A) HepAD38 cells were sequentially treated with 2 mM of PFA and 1 μM of ETV, 2.5 μM of Bay 41–4109, 1 μM of GLS4 or 5 μM of ENAN-34017 and harvested as the schedule depicted. The cytoplasmic core DNA (B), cccDNA and DP-rcDNA (C) were analyzed Southern blot hybridization. The relaxed circular (RC) DNA, double-stranded linear (DSL) DNA and full-length single stranded (SS) DNA species are indicated. For cccDNA and DP-rcDNA analysis, before loading, Hirt DNA were denatured at 88°C for 5 min and followed by digestion with EcoRI. (TIF) [file ppat.1006658.s006.tif]

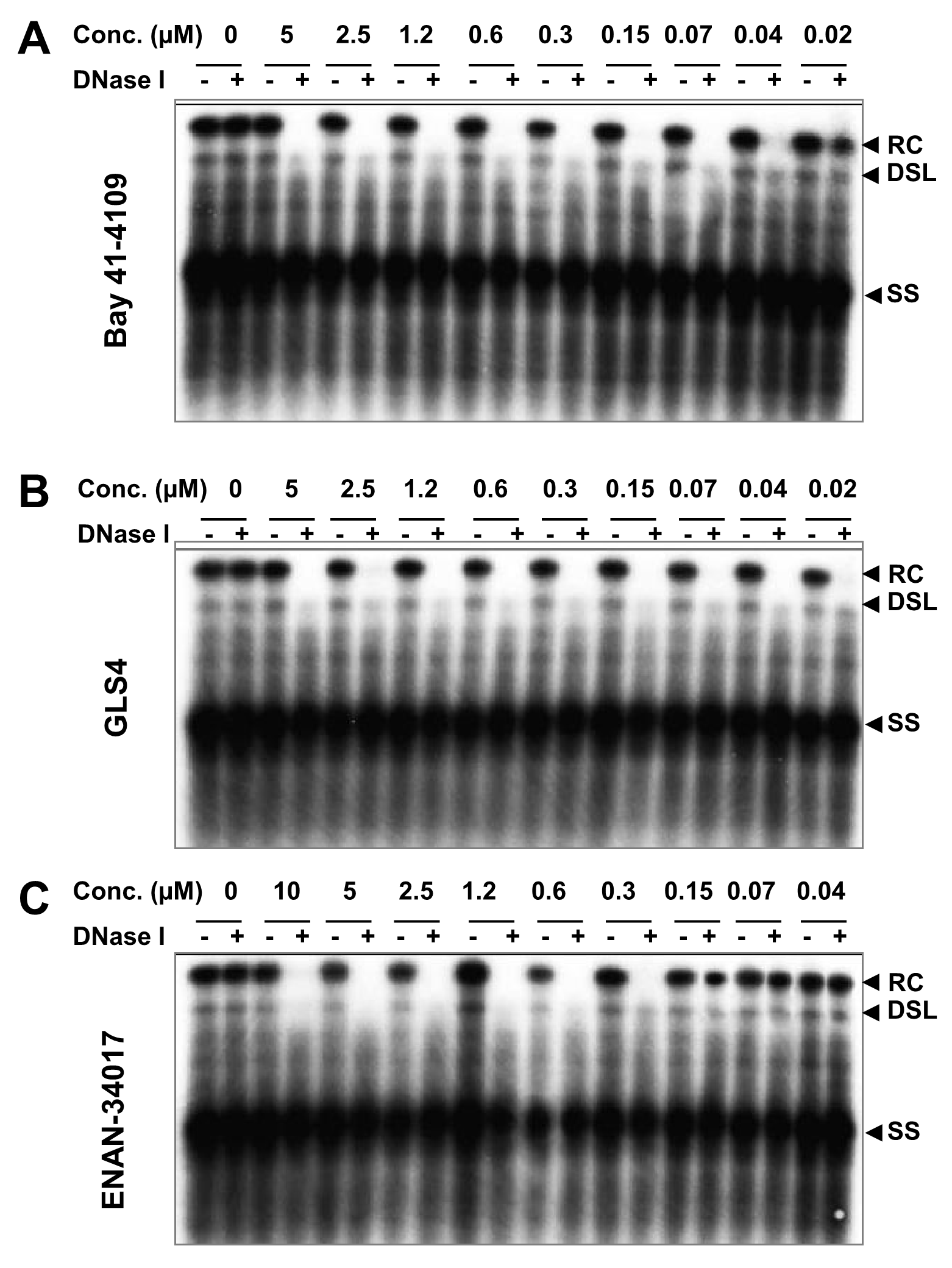

Supplement: S7 Fig — HBV capsids prepared from HepAD38 cells were incubated with the indicated concentrations of Bay 41–4109 (A), GLS4 (B) or ENAN-34017 (C) in reactions containing 150 mM NaCl, 50 mM Tris-HCl, pH8.0, 10 mM MgCl2, 1 mM DTT and 0.1% NP-40 at 37°C for 16 h and then left untreated or treated with 10 μg/ml DNase I for 30 mins. Viral DNA was extracted and resolved by agarose gel electrophoresis and detected by Southern blot hybridization with full-length riboprobe recognizing minus strand DNA. The relaxed circular (RC) DNA, double-stranded linear (DSL) DNA and full-length single stranded (SS) DNA species are indicated. (TIF) [file ppat.1006658.s007.tif]

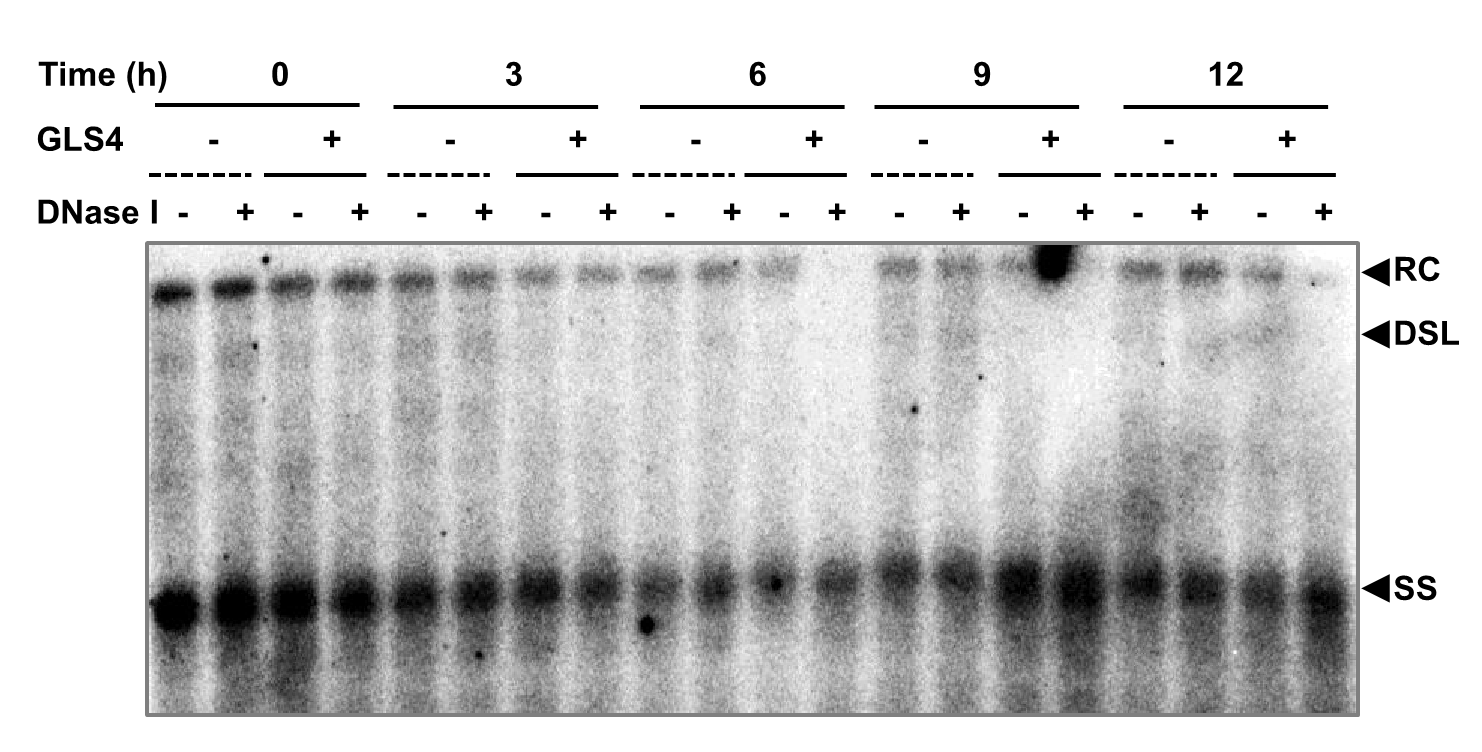

Supplement: S8 Fig — HepAD38 cells were cultured in tet-free medium for six days and then mock-treated or treated with 1μM GLS4 for the indicated period of time. The cells were harvested and cytoplasmic DNA was extracted without or with prior treatment of cell lysates with 10 μg/ml of DNase I for 30 mins. The viral DNA were resolved in agarose gel and detected by Southern blot hybridization with full-length riboprobe recognizing minus strand DNA. The relaxed circular (RC) DNA, double-stranded linear (DSL) DNA and full-length single stranded (SS) DNA species are indicated. (TIF) [file ppat.1006658.s008.tif]

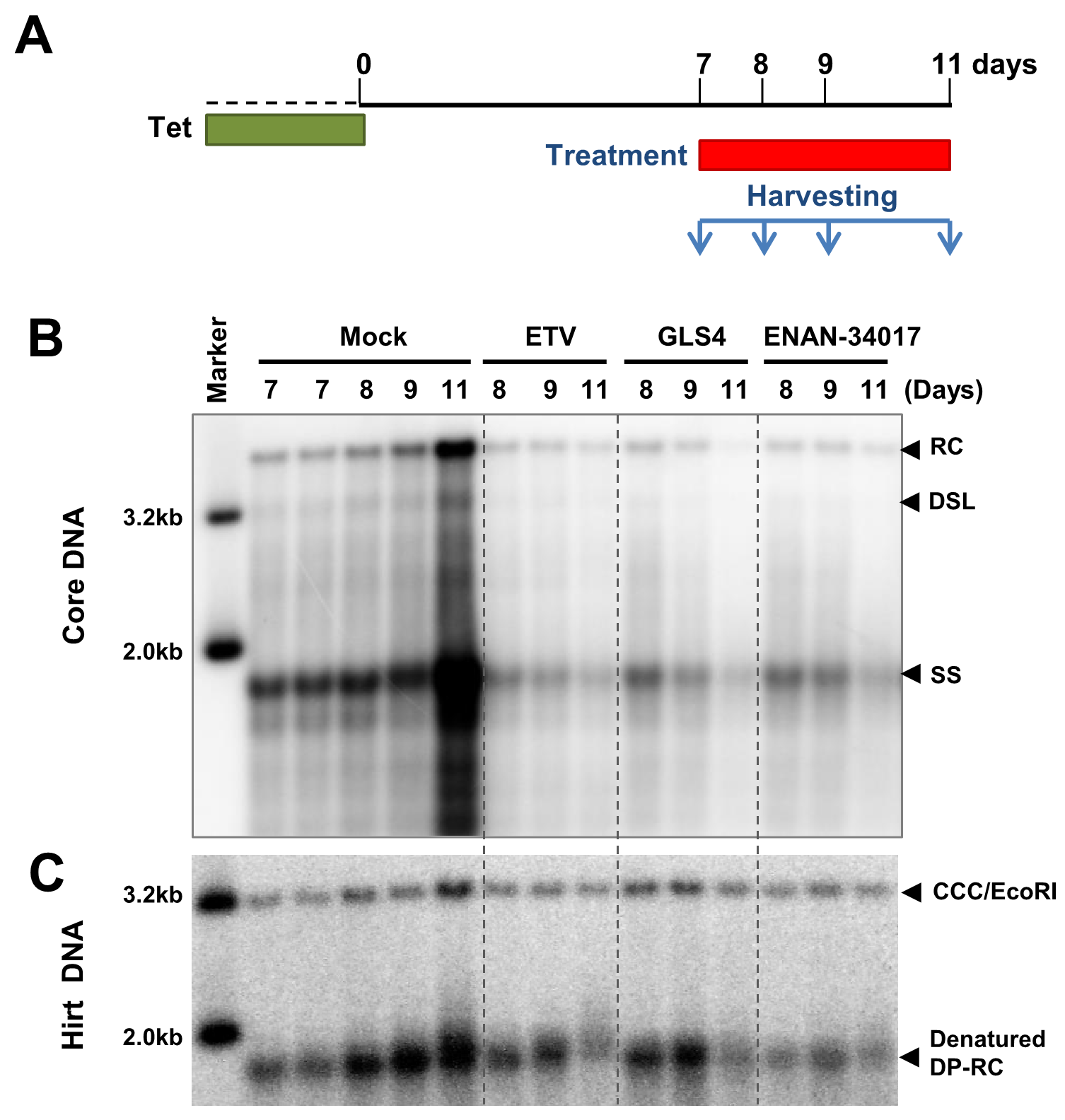

Supplement: S9 Fig — (A) HepAD38 cells were cultured in the absence of tetracycline for 7 days to allow HBV pgRNA transcription, DNA replication and cccDNA synthesis to occur. The cells were then mock-treated or treated with ETV (1 μM), GLS4 (1 μM) or ENAN-34017 (5 μM). Cells were harvested on day 1, 2, 4 of treatment. Viral core DNA (B) and Hirt DNA (C) were extracted and analyzed Southern blot hybridization with a riboprobe specific to negative strand DNA. The relaxed circular (RC) DNA, double-stranded linear (DSL) DNA and full-length single stranded (SS) DNA species are indicated. For cccDNA and DP-rcDNA analysis, before loading, Hirt DNA were denatured at 88°C for 5 min and followed by digestion with Eco RI. 3.2 kb and 2.0 kb HBV DNA served as a molecular weight markers. (TIF) [file ppat.1006658.s009.tif]
